# Control regimes of nitrate air pollution reconciled by multiphase dissociation equilibrium

**DOI:** 10.1093/nsr/nwag481

**Published:** 2026-08-11

**Authors:** Guangjie Zheng, Guannan Geng, Yuexuanzi Wang, Yuxi Liu, Ruilin Wan, Yihao Wang, Hongli Wang, Xiue Shen, Jingkun Jiang, Qiang Zhang, Kebin He, Yafang Cheng, Hang Su

**Affiliations:** State Key Laboratory of Regional Environment and Sustainability, School of Environment, Tsinghua University, Beijing 100084, China; Aerosol Chemistry Department, Max Planck Institute for Chemistry, Mainz 55128, Germany; State Key Laboratory of Regional Environment and Sustainability, School of Environment, Tsinghua University, Beijing 100084, China; Ministry of Education Key Laboratory for Earth System Modeling, Department of Earth System Science, Tsinghua University, Beijing 100084, China; State Key Laboratory of Regional Environment and Sustainability, School of Environment, Tsinghua University, Beijing 100084, China; Key Laboratory of Environmental Pollution and Greenhouse Gases Co-control, Ministry of Ecology and Environment, Chinese Academy of Environmental Planning, Beijing 100041, China; State Key Laboratory of Regional Environment and Sustainability, School of Environment, Tsinghua University, Beijing 100084, China; State Key Laboratory of Regional Environment and Sustainability, School of Environment, Tsinghua University, Beijing 100084, China; State Environmental Protection Key Laboratory of the Cause and Prevention of Urban Air Pollution Complex, Shanghai Academy of Environmental Sciences, Shanghai 200233, China; Beijing Municipal Ecological and Environmental Monitoring Center, Beijing 100048, China; State Key Laboratory of Regional Environment and Sustainability, School of Environment, Tsinghua University, Beijing 100084, China; Ministry of Education Key Laboratory for Earth System Modeling, Department of Earth System Science, Tsinghua University, Beijing 100084, China; State Key Laboratory of Regional Environment and Sustainability, School of Environment, Tsinghua University, Beijing 100084, China; Aerosol Chemistry Department, Max Planck Institute for Chemistry, Mainz 55128, Germany; Aerosol Chemistry Department, Max Planck Institute for Chemistry, Mainz 55128, Germany; Institute of Atmospheric Physics, Chinese Academy of Sciences, Beijing 100029, China

**Keywords:** nitrate air pollution, gas-particle partitioning, multiphase processes, aerosol acidity, sulfate-nitrate interactions

## Abstract

Particulate nitrate is a major component of haze pollution, yet substantial debate exists regarding its effective control policies. Here, we develop a multiphase dissociation equilibrium equation, based on which the influence of meteorological conditions and chemical profiles can be decoupled, and the analytical expression of nitrate sensitivity to different species can be derived. With this framework, four nitrate control regimes are identified, namely the nitric-acid-control, ammonia-control, transition, and meteorology regimes. The framework also explains the trade-offs between sulfate and nitrate, and elucidates the influence of sulfate and non-volatile cations on the regime transitions. Across most Northern Hemisphere continental regions, and nearly all of China, particulate nitrate is generally more sensitive to nitric acid than to ammonia due to the universal NH_3_ excess. The framework provides transparent criteria for interpreting nitrate sensitivity and control regime classifications, serving as an important scientific basis for nitrate control policies and reactive nitrogen interactions.

## Teaser

A novel thermodynamic framework decouples meteorological and chemical influences, providing transparent criteria to classify nitrate control regimes, and revealing that nitrogen oxide reduction is highly effective in typical nitrate polluted regions.

## INTRODUCTION

Atmospheric nitrate has drawn growing attention due to its essential role in haze pollution, nitrogen deposition, and climate change [1–8]. Especially in China, nitrate has become a leading component of haze pollution, generally accounting for ∼30% of the PM_2.5_ mass, greatly surpassing the contribution of sulfate since the ‘Clean Air Actions’ [2,5,9,10]. Unlike sulfate, nitrate is more volatile, and gas-particle partitioning can sometimes be more decisive than chemical production in determining particulate nitrate levels [2,11–15]. As ammonia strongly affects nitrate partitioning, some studies suggest that NH_3_ reduction is more effective than NOx reduction for nitrate control [2,13,16–19], while some others draw opposite conclusions [10,20–22]. Furthermore, large SO_2_ reductions have been proposed to enhance the particle-phase partitioning of nitrate in China [18,23,24], while no such pattern is observed in the southeastern USA (SE-US) [25].

These discrepancies arise from the complex processes involved in nitrate partitioning, including acid dissociation equilibrium, gas–liquid partitioning, and charge balance constraints, etc. These processes are jointly influenced by aerosol acidity (or pH), non-ideality, aerosol water contents (*L*_w_), temperature (*T*), and the particle-phase chemical compositions, etc. (see our summary in Supplementary Text S1 and Fig. S1), resulting in strong nonlinearity and substantial variability across regions and timescales [11,12,26,27]. Previous studies have substantially improved our understanding of nitrate gas-particle partitioning thermodynamics by identifying aerosol pH and *L*_w_ as key variables [28]. However, aerosol pH is strongly driven by *L*_w_ in multiphase buffered system [29], rather than being an independent variable. Besides *L*_w_, aerosol pH is further inherently coupled to a complex interplay of aerosol composition and meteorological conditions (Fig. S1). Therefore, the pH–*L*_w_ framework cannot readily compare nitrate control regimes across distinct chemical and climatic environments. Furthermore, existing frameworks lack a straightforward analytical expression to unify the roles of sulfate, crustal cations, and meteorology.

Here, we develop a multiphase dissociation equilibrium equation that decouples meteorological and chemical influences by eliminating the explicit dependence on pH. With this framework, we derive the analytical expression of nitrate sensitivity to different species, which provides a physically transparent criterion for nitrate control regime classifications. Moreover, this framework enables direct comparison of regime distributions and straightforward identification of dominant factors across different scenarios. Our results provide practical quantitative criteria for air pollution policies, reconciling the debate on nitrate control measures, with broader implications for global nitrogen deposition, climate studies, and ammonia energy assessments.

## RESULTS AND DISCUSSION

### Four types of nitrate control regimes

To better understand the controlling factors of particulate nitrate (NO_3_^−^), we conducted a series of sensitivity tests with the ISORROPIA v2.3 model [30] based on worldwide observations (Method M1) [31–42]. Total ammonia (NH_3_^T^) and total nitrate (HNO_3_^T^) in both gas and aqueous phases are used to represent NH_3_ and NOx emission changes, respectively, while sulfate is assumed to be proportional to SO_2_ emissions [2,42]. The NO_3_^−^ is defined as sensitive to species X if the absolute value of sensitivity *S*(X) = ∂[NO_3_^−^]/∂[X] is above 0.1, that is, a unit change in X corresponds to at least 10% of the corresponding change in NO_3_^−^ [28]. Note that [X] represents the atmospheric molar concentration of species X (unit: μmol m^−3^) hereinafter.

**Figure 1.**
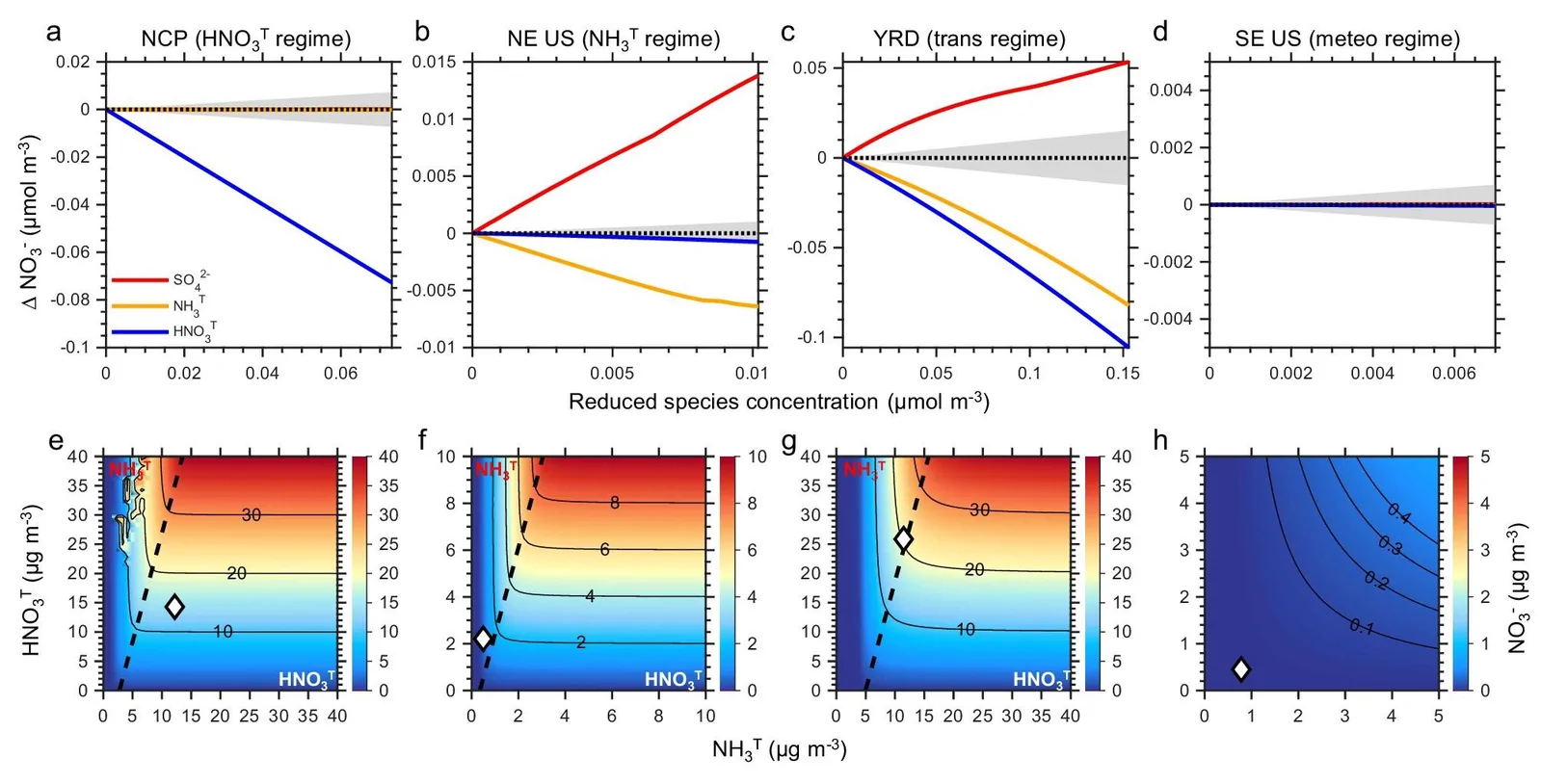
Contrasting sensitivities of particulate nitrate NO_3_^−^ to variations in NH_3_^T^, HNO_3_^T^, and SO_4_^2−^ under different scenarios. The four base scenarios (starting points of panels a–d, and diamond markers in panels e–h) represent observation-derived conditions in the SE-US, NE-US, the YRD, and the NCP region of China, respectively (see detailed scenario setting in Table S1). The results are based on ISORROPIA v 2.3 model calculations, by varying the target species only while keeping all the other variables the same. In panels (a–d), the *x* axis refers to the reduced NH_3_^T^, HNO_3_^T^, or SO_4_^2−^ concentrations from the base scenarios, while the different lines show the corresponding NO_3_^−^ changes. The maximum reduction degree (i.e. end point of *x* axis) in each panel is set to be 98% the molar concentration of the limiting species, that is, the species with minimal molar concentrations among NH_3_^T^, HNO_3_^T^, and SO_4_^2−^. The gray-shaded areas indicate the insensitive range, when the absolute value of nitrate sensitivity is below 0.1. In panels (e and f), the variation ranges of NH_3_^T^ and HNO_3_^T^ are manually set to show the general patterns, when the sensitivities are generally parallel to *x*- or *y*-axis (e.g. panel e–g) or when over 10 times of the limiting species is added to the original system (e.g. HNO_3_^T^ in panel h).

We first investigated the NO_3_^−^ sensitivity to NH_3_^T^ and HNO_3_^T^, which are the focus of previous debate. Four representative scenarios were found in the tests (see Table S1) [31–34], which covered all possible *S*(NH_3_^T^) and *S*(HNO_3_^T^) combinations (Fig. 1). That is, when NO_3_^−^ is sensitive to: (i) HNO_3_^T^ only, like the winter North China Plain (NCP, Fig. 1a); (ii) NH_3_^T^ only, like the northeastern United States (NE-US, Fig. 1b), (iii) both NH_3_^T^ and HNO_3_^T^, like the polluted autumn Yangtze River Delta scenario (YRD, Fig. 1c); and (iv) neither NH_3_^T^ nor HNO_3_^T^, like the SE-US scenario (Fig. 1d). For simplicity, we denote these four regimes as ‘HNO_3_^T^’, ‘NH_3_^T^’, ‘Trans’ (short for ‘Transition’), and ‘Meteo’ (short for meteorology-limited) regimes, respectively. Clearly, NH_3_ emission control is useful only under the ‘NH_3_^T^’ and ‘Trans’ regimes, and NOx control is effective only under the ‘HNO_3_^T^’ and ‘Trans’ regimes. In comparison, neither of them is effective under the ‘Meteo’ regime. We also show that the local sensitivities *S*(X) at the origin point (i.e. the starting point in Fig. 1) could well indicate the more effective control pathway overall. The sensitivity to different species (i.e. corresponding slopes in Fig. 1) remained stable before the limiting species was nearly exhausted. Consequently, nitrate decreases more rapidly if the more sensitive species is controlled first.

Another typical way to identify the HNO_3_^T^- vs. NH_3_^T^-regime is the classic plot of NO_3_^−^ against HNO_3_^T^ and NH_3_^T^ concentrations (Fig. 1e–h). However, the regime transition lines (black dashed lines in Fig. 1e–g) as well as the shape of the NO_3_^−^ isolines varied significantly across scenarios, precluding direct comparison in a unified plot and complicating regime determination. In addition, the sensitivities of nitrate to sulfate (Fig. 1a–d) and temperature (Fig. S2) also differed significantly among scenarios, which cannot be illustrated or explained in plots like Fig. 1e–h. Moreover, such plots do not reveal the underlying principles of regime transitions.

### Multiphase dissociation equilibrium of ammonium nitrate

**Figure 2.**
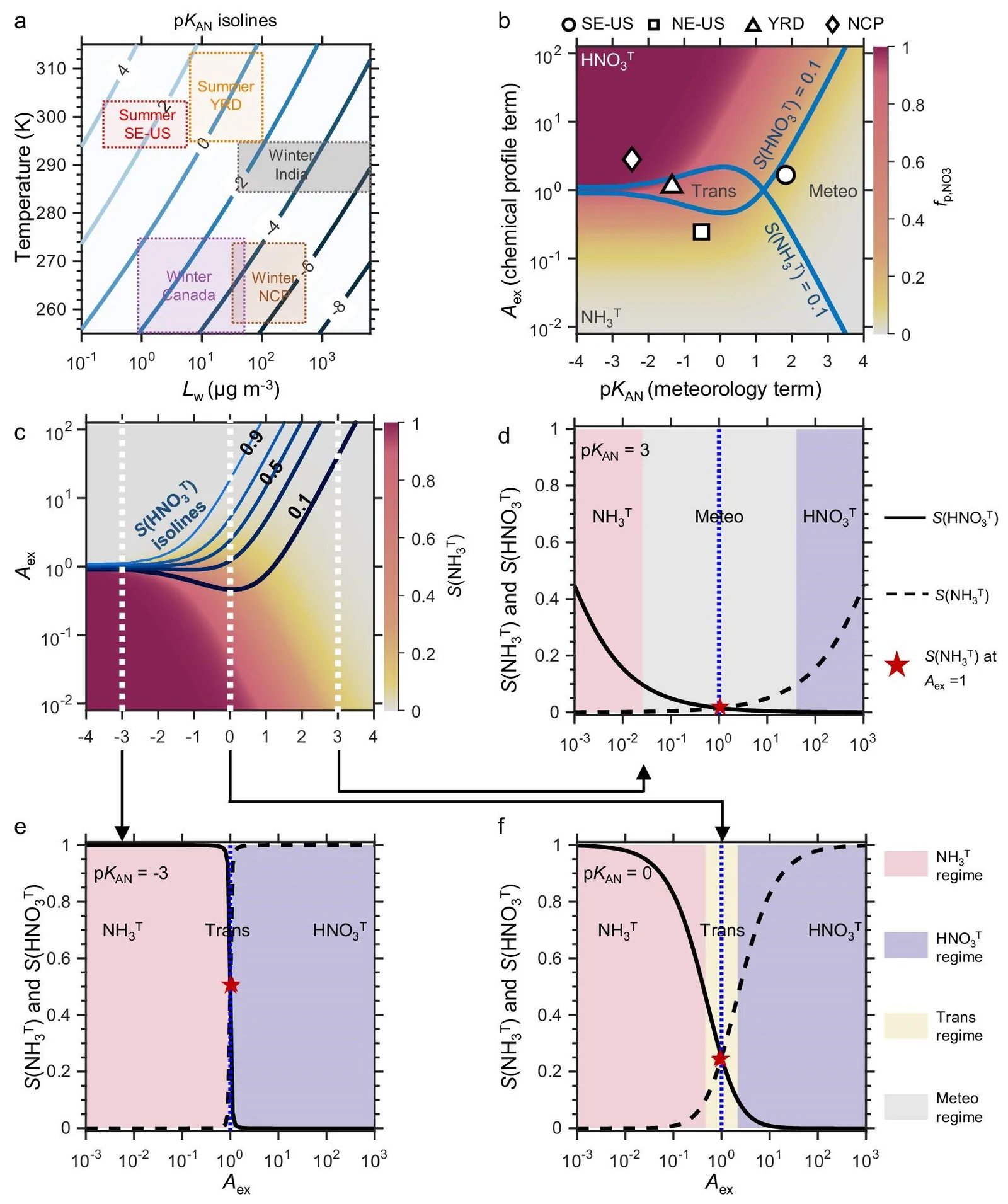
Regime classification according to the sensitivity of particulate nitrate NO_3_^−^ to emissions. (a) Dependence of p*K*_AN_ on *L*_w_ and temperature at a typical γ^2^_AN_ = 0.05 (see Supplementary Information S2 for details). The squares indicate the rough *L*_w_ and temperature ranges based on long-term observations at typical regions, as detailed in Method M1 and Table S3. (b and c) Dependence of (b) *f*_p, NO3_, (c) sensitivity of nitrate to total ammonia *S*(NH_3_^T^) = ∂[NO_3_^−^]/∂[NH_3_^T^] and total nitrate *S*(HNO_3_^T^) = ∂[NO_3_^−^]/∂[HNO_3_^T^] on p*K*_AN_ and *A*_ex_ for the case when free sulfate is zero (i.e. *S*_f_ = 0). In panel (b), the dashed lines indicate the borders of regime classifications, that is, *S*(NH_3_^T^) = 0.1 and *S*(HNO_3_^T^) = 0.1, respectively. The shapes indicate the four scenarios as shown in Fig. 1. In panel (c), the colorbar and isolines represent *S*(NH_3_^T^) and *S*(HNO_3_^T^), respectively. (d–f) Dependence of *S*(NH_3_^T^) and *S*(HNO_3_^T^) with *A*_ex_ at three p*K*_AN_ levels, which are cross-sections of the three dashed lines in panel (c). The red stars indicate the *S*(NH_3_^T^) (which is equal to *S*(HNO_3_^T^)) at *A*_ex_ = 1. Nitrate control regime changes along *A*_ex_ variations are labelled.

To identify dominant drivers of the nitrate control regime and the underlying mechanisms, we examined the influence of aerosol acidity on nitrate partitioning, which is the key nexus of emissions, meteorology, and nitrate concentration (Supplementary Information S1, Fig. S1) [20,28,43]. However, by applying the recently proposed multiphase buffer theory [29], we found that the explicit dependence on pH can be eliminated within the ammonium–nitrate multiphase equilibrium framework, owing to the counteracting effects of pH on the NH_4_^+^/NH_3_ and HNO_3_/NO_3_^−^ buffer systems (see Method M2 and Supplementary Text S3 for details). For most non-coastal continental urban areas, the nitrate partitioning is governed by the multiphase dissociation equilibrium of ammonium nitrate:


(1a) \begin{eqnarray*}
{\mathrm{N}}{{\mathrm{H}}}_3({\mathrm{g}}) &+& {\mathrm{HN}}{{\mathrm{O}}}_3({\mathrm{g}}) \rightleftharpoons {\mathrm{N}}{{\mathrm{H}}}_4^ {\,\,+} \left( {{\mathrm{aq}}} \right)\\
&+& {\mathrm{N}}{{\mathrm{O}}}_3^ {\,\,-} \left( {{\mathrm{aq}}} \right),
\end{eqnarray*}

with the equilibrium constant *K*_AN_ being


(1b) \begin{eqnarray*}
K_{{\mathrm{AN}}}^{} &=& \frac{{[{\mathrm{N}}{{\mathrm{O}}}_{\mathrm{3}}^ {\,\,-} \left( {{\mathrm{aq}}} \right)][{\mathrm{N}}{{\mathrm{H}}}_{\mathrm{4}}^{\mathrm{\,\, + }}{\mathrm{(aq)]}}}}{{{\mathrm{[HN}}{{\mathrm{O}}}_{\mathrm{3}}{\mathrm{(g)][N}}{{\mathrm{H}}}_{\mathrm{3}}{\mathrm{(g)]}}}}\\
&=& \frac{{{K}_{{\mathrm{a,HNO3}}}{H}_{{\mathrm{HNO3}}}{H}_{{\mathrm{NH3}}}}}{{{K}_{{\mathrm{a,NH3}}}}}{\left(\frac{{\ R\ T\ {L}_{\mathrm{w}}}}{{{\rho }_w{\gamma }_{{\mathrm{AN}}}}}\right)}^2,
\end{eqnarray*}

where *K*_a,i_ and *H*_i_ are the acid dissociation constant and Henry’s law coefficient for species *i*, respectively (see Table S2); *L*_w_ is the aerosol water content, *ρ*_w_ is the liquid water density, *R* is the gas constant, *T* is the absolute temperature, and γ_AN_ is the activity coefficient of ammonium nitrate defined as (γ_NH4+_γ_NO3-_)^0.5^. *K*_AN_ depends only on *T* and *L*_w_ at given γ_AN_ levels. Because *L*_w_ is primarily determined by PM_2.5_ concentrations, *K*_AN_ largely represents the influence of meteorological conditions (temperature, humidity, and dilution conditions). The negative logarithm of *K*_AN_, p*K*_AN_ = −log(*K*_AN_), can vary up to ∼12 units under ambient-relevant *L*_w_ and temperature conditions (Fig. 2a). Low temperature and/or high *L*_w_ (e.g. winter NCP and India) correspond to high *K*_AN_ (low p*K*_AN_), while high temperature and/or low *L*_w_ (e.g. summer SE-US) correspond to high p*K*_AN_ levels. In comparison, p*K*_AN_ fluctuation due to chemical profiles through γ_AN_ and hygroscopicity is typically within 1 unit, much smaller than that from meteorological conditions. Detailed analysis of p*K*_AN_ uncertainties and their impacts is discussed in Supplementary Information S2 and Fig. S3.

Combining this equilibrium with other constraints, we simplify the complex picture of nitrate partitioning (Fig. S1) into a function of


(2a) \begin{eqnarray*}
K_{{\mathrm{AN}}}^{}({A}_{{\mathrm{ex}}} - {f}_{{\mathrm{p,NO3}}}) = \frac{{{f}_{{\mathrm{p,NO3}}}}}{{1 - {f}_{{\mathrm{p,NO3}}}}}({f}_{{\mathrm{p,NO3}}} + {S}_{\rm f}).
\end{eqnarray*}

Here, *f*_p, NO3_ is the molar fraction of particle-phase nitrate (i.e. [NO_3_^−^]/[HNO_3_^T^]), *S*_f_ is the molar ratio of free sulfate to total nitrate as


(2b) \begin{eqnarray*}
{{\mathit{S}}}_{\mathrm{f}} = \left( {2\left[ {{\mathrm{S}}{{\mathrm{O}}}_4^{\,\,2 - }\left( {{\mathrm{aq}}} \right)} \right] - \left[ {{\mathrm{NVCs}}} \right]} \right)/\left[ {{\mathrm{HN}}{{\mathrm{O}}}_3^{\mathrm{\,\,T}}} \right],
\end{eqnarray*}

where NVCs represent the total non-volatile cations defined as


(2c) \begin{eqnarray*}
\left[ {{\mathrm{NVCs}}} \right] &=& 2\left[ {{\mathrm{C}}{{\mathrm{a}}}^{2 + }\left( {{\mathrm{aq}}} \right)} \right] + 2\left[ {{\mathrm{M}}{{\mathrm{g}}}^{2 + }\left( {{\mathrm{aq}}} \right)} \right] \\
&& + \left[ {{{\mathrm{K}}}^ + \left( {{\mathrm{aq}}} \right)} \right] + \left[ {{\mathrm{N}}{{\mathrm{a}}}^ + \left( {{\mathrm{aq}}} \right)} \right],
\end{eqnarray*}

and *A*_ex_ is the molar ratio of excess ammonia to total nitrate as


(2d) \begin{eqnarray*}
{{\mathit{A}}}_{{\mathrm{ex}}} &=& {{\mathit{R}}}_{{\mathrm{A}}/{\mathrm{N}}}-{{\mathit{S}}}_{\mathrm{f}} = \left( \left[ {{\mathrm{N}}{{\mathrm{H}}}_3^{\mathrm{\,\,T}}} \right] + \left[ {{\mathrm{NVCs}}} \right]\right.\\
&& \left.- 2\left[ {{\mathrm{S}}{{\mathrm{O}}}_4^{\,\,2 - }\left( {{\mathrm{aq}}} \right)} \right] \right)/\left[ {{\mathrm{HN}}{{\mathrm{O}}}_3^{\mathrm{\,\,T}}} \right],
\end{eqnarray*}

where *R*_A/N_ is the molar ratio of NH_3_^T^ to HNO_3_^T^ (i.e. [NH_3_^T^]/[HNO_3_^T^]).

Equation 2 quantitatively describes how meteorological conditions and chemical profiles jointly govern nitrate partitioning (*f*_p, NO3_). Meteorological conditions are represented by *K*_AN_, while chemical profiles are indicated by *S*_f_ and *A*_ex_. The *S*_f_ represents the contribution of non-volatile components, while *A*_ex_ quantifies the degree of ammonia excess (Method M2 and Supplementary Text S3).

From Equation 2, *f*_p,NO3_ can be solved, and the analytical expression of nitrate sensitivities to different chemical components and meteorological parameters are derived (see Table 1 and Supplementary Information S3). In particular, we show that


(3) \begin{eqnarray*}
{\mathit{S}}( {{\mathrm{N}}{{\mathrm{H}}}_3^{\mathrm{\,\,T}}} ) = {\mathit{S}}( {{\mathrm{HN}}{{\mathrm{O}}}_3^{\mathrm{\,\,T}}}),{\mathrm{when}}\,{{\mathit{A}}}_{{\mathrm{ex\ }}} = 1.
\end{eqnarray*}

**Table 1.** Expressions the sensitivities of nitrate to different chemical species X, *S*(X) = ∂[NO_3_^−^]/∂[X].

**Variable and definitions**	**Solution expressed in chemical species** ^a^	**Analytical solution**	**Equation No.**
*S*(HNO_3_^T^)=∂[NO_3_^−^]/∂[HNO_3_^T^]	${{\mathrm{([HN}}{{\mathrm{O}}}_{\mathrm{3}}{\mathrm{(g)] }}{R}_{\textit{chem}})}^{ - 1}$	${\Big(1 - \frac{1}{{{K}_{{\mathrm{AN}}}}} + \frac{{{R}_{{\mathrm{A/N}}}{A}_{{\mathrm{ex}}}}}{{{K}_{{\mathrm{AN}}}{{({A}_{{\mathrm{ex}}} - {f}_{_{{\mathrm{p,NO3}}}})}}^2}}\Big)}^{ - 1}$	(4)
*S*(NH_3_^T^)=∂[NO_3_^−^]/∂[NH_3_^T^]	${({\mathrm{[N}}{{\mathrm{H}}}_{\mathrm{3}}{\mathrm{(g)] }}{R}_{\textit{chem}})}^{ - 1}$	${\Big(1 - \frac{1}{{{K}_{{\mathrm{AN}}}}} + \frac{{1 + {S}_f}}{{{K}_{{\mathrm{AN}}}{{(1 - {f}_{_{{\mathrm{p,NO3}}}})}}^2}}\Big)}^{ - 1}$	(5)
*S*(SO_4_^2−^)=∂[NO_3_^−^]/∂[SO_4_^2−^]	$- 2*{\Big(\frac{{{\mathrm{[N}}{{\mathrm{H}}}_{\mathrm{3}}{\mathrm{(g)]}}[{\mathrm{N}}{{\mathrm{H}}}_{\mathrm{4}}^{\mathrm{\,\, + }}{\mathrm{(aq)]}}}}{{[{\mathrm{N}}{{\mathrm{H}}}_{\mathrm{3}}^{\mathrm{T}}{\mathrm{]}}}}{R}_{\textit{chem}}{\mathrm{\Big)}}}^{{\mathrm{ - 1}}}$	$- 2*{\Big(1 + \frac{{{K}_{{\mathrm{AN}}}{R}_{{\mathrm{A/N}}}}}{{{{({K}_{{\mathrm{AN}}} + (1 - {K}_{{\mathrm{AN}}}){f}_{p{\mathrm{, NO3}}})}}^2}}\Big)}^{ - 1}$	(6)
*S*(NVCs)= ∂[NO_3_^−^]/∂[NVCs]	${\Big(\frac{{{\mathrm{[N}}{{\mathrm{H}}}_{\mathrm{3}}{\mathrm{(g)]}}[{\mathrm{N}}{{\mathrm{H}}}_{\mathrm{4}}^{\mathrm{\,\, + }}{\mathrm{(aq)]}}}}{{[{\mathrm{N}}{{\mathrm{H}}}_{\mathrm{3}}^{\mathrm{T}}{\mathrm{]}}}}{R}_{\textit{chem}}\Big)}^{ - 1}$	${\Big(1 + \frac{{{K}_{{\mathrm{AN}}}{R}_{{\mathrm{A/N}}}}}{{{{({K}_{{\mathrm{AN}}} + (1 - {K}_{{\mathrm{AN}}}){f}_{p{\mathrm{, NO3}}})}}^2}}\Big)}^{ - 1}$	(7)

All variables are with units of unity. Chemical species shown indicate their atmospheric molar concentrations (unit: μmol m^−3^). The definition of *R*_chem_ is noted below. Other symbols are defined in the text as: *R*_A/N_ = [NH_3_^T^]/[HNO_3_^T^], *f*_p, NO3_ = [NO_3_^−^]/[HNO_3_^T^], *S*_f_ = (2[SO_4_^2−^(aq)]−[NVCs])/[HNO_3_^T^], and *A*_ex_ = *R*_A/N_—*S*_f_.

a The term *R*_chem_ is defined as: *R*_chem_ = [NH_3_(g)]^−1^ +[HNO_3_(g)]^−1^ + [NH_4_^+^(aq)]^−1^ + [NO_3_^−^(aq)]^−1^.

This actually corresponds to when the total potential cations (i.e. [NVCs] + [NH_3_^T^]) equals the total potential anions (i.e. 2[SO_4_^2−^] + [HNO_3_^T^]). At higher *A*_ex_ levels, ammonia is in excess and *S*(NH_3_^T^) < *S*(HNO_3_^T^), whereas at lower *A*_ex_ levels, the reverse is true.

Several constraints must be satisfied for the above framework (Equations 2–7) to be applicable. First, the nitrate exists dominantly as ammonium-nitrate, namely when (i) total chloride contributes <10% of total anions; (ii) non-volatile cations are smaller than total anions, and (iii) non-volatile anions (mainly sulfate) are smaller than total cations (Method M2). Second, the gas-particle equilibrium is a reasonable approximation. For this, we selected conditions where RH is over 40% and for fine-mode particles only (Method M2). Most of the non-coastal continental urban areas satisfy these conditions (Fig. S4a), and for these areas, *f*_p,NO3_ solved by Equation 2 agrees well with thermodynamic models (*R*^2^∼1.0; see Fig. S4b).

### Nitrate control regimes explained by the multiphase dissociation equilibrium

Figure 2b–d shows the nitrate partitioning and nitrate sensitivity to NH_3_^T^ and HNO_3_^T^ as derived from Equations 4 to 5, for the case when free sulfate is zero (i.e. *S*_f_ = 0). The two blue dash lines represent isolines of *S*(NH_3_^T^) = 0.1 and *S*(HNO_3_^T^) = 0.1, respectively, which are the boundaries of the four aforementioned regimes (Fig. 1). The *K*_AN_ largely represents the influence of meteorological conditions, while *A*_ex_ represents the influence of chemical profiles.

Under clean and hot conditions with high p*K*_AN_ levels of 2∼4, the meteorology greatly favors the gas-phase partitioning of nitrate, resulting in low *f*_p,NO3_ (Fig. 2b). Correspondingly, NH_3_^T^ to HNO_3_^T^ variations would be reflected largely in the gas-phase concentrations, rather than in NO_3_^−^. This is the meteorology-limited regime with low *S*(NH_3_^T^) and *S*(HNO_3_^T^) (Fig. 2b–d). In comparison, under polluted and cold conditions with low p*K*_AN_ levels (e.g. below −2), nitrate preferentially partitions into the particle phase, and the *f*_p,NO3_ is largely controlled by the limiting species. When NH_3_^T^ is in excess (i.e. *A*_ex_ > 1 here), nearly all HNO_3_^T^ resides in the particle phase (*f*_p,NO3_∼1; Fig. 2b), and NO_3_^−^ concentration is determined by the available HNO_3_^T^. Therefore, *S*(HNO_3_^T^) is high while *S*(NH_3_^T^) is low (Fig. 2c, e), which is the ‘HNO_3_^T^’ regime. Similarly, the ‘NH_3_^T^’ regime occurs when NH_3_ is the limiting species (i.e. *A*_ex_ < 1). Note that the regime transition can be quite sharp around *A*_ex_ = 1 (Fig. 2e), indicating the dominant influence of chemical profiles at low p*K*_AN_ levels. At moderate p*K*_AN_ (e.g. −2 to 2), both meteorology and chemical profiles matter, and the ‘Trans’ regimes would occur under comparable availability of NH_3_^T^ and HNO_3_^T^ (Fig. 2b, c, and f). Note that at a given p*K*_AN_ level, whether the transition or meteorology-limited regime would occur depends on the sensitivity at *A*_ex_ = 1, when *S*(NH_3_^T^) = *S*(HNO_3_^T^) (red stars in Fig. 2d–f). The meteorology-limited regime occurs if this value is below 0.1; otherwise the ‘Trans’ regime applies. The regime classifications agree well with the patterns observed in Fig. 1 (see corresponding distributions in Fig. 2b). Moreover, the varying shapes of NO_3_^−^ isolines (Fig. 1e–h) are well explained by the different p*K*_AN_ levels (Fig. 2d–f).

### Influence of non-volatile sulfate and crustal ions

**Figure 3.**
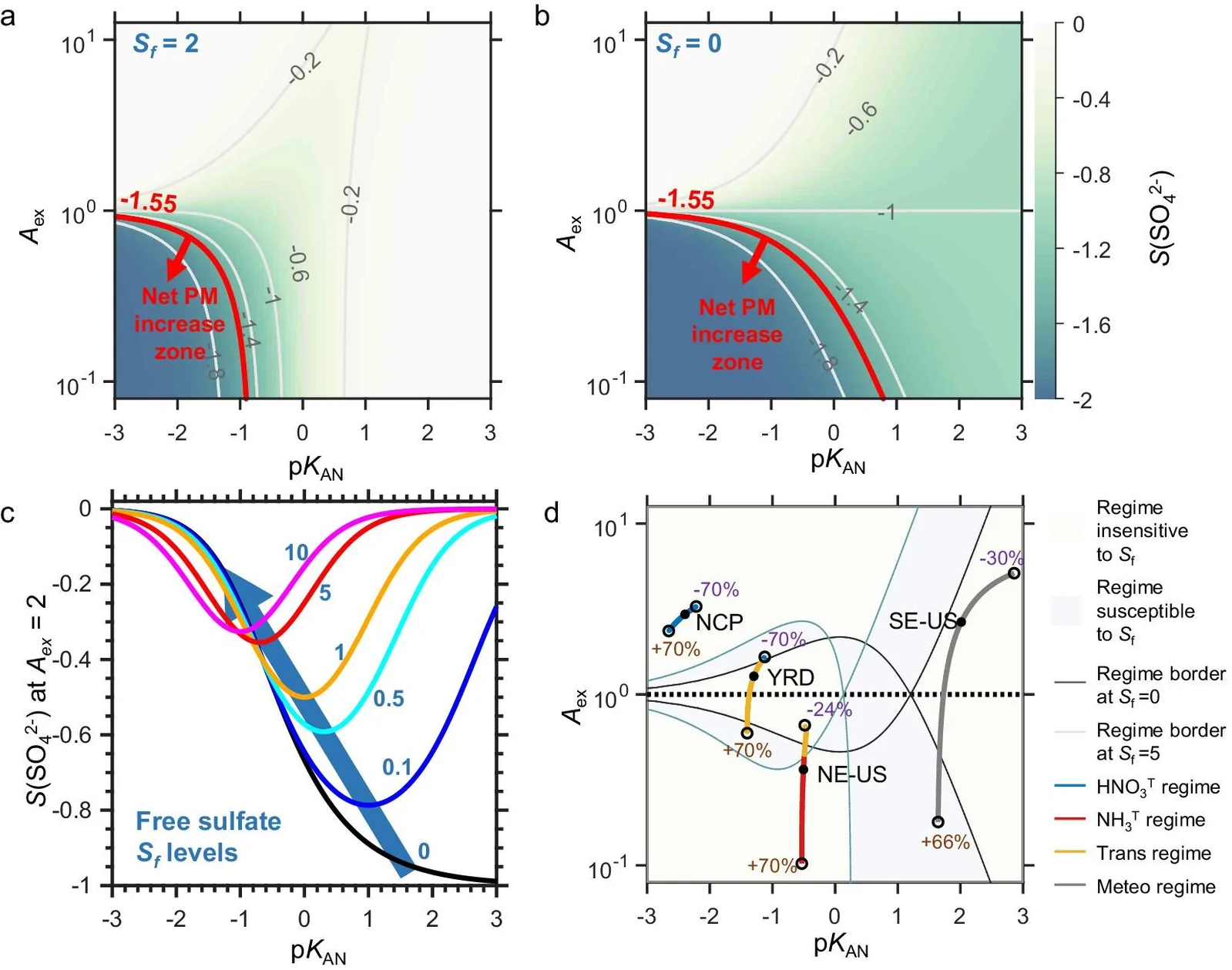
Influence of SO_4_^2−^ on nitrate. (a, b) Dependence of nitrate sensitivity to sulfate under different meteorological conditions (as indicated by p*K*_AN_ levels) and chemical profiles (as indicated by the *A*_ex_), at free sulfate *S*_f_ levels of (a) two and (b) zero. (c) Dependence of *S*(SO_4_^2−^) on p*K*_AN_ levels at different *S*_f_ levels, when *A*_ex_ is fixed at 2. (d) Influence of sulfate on the nitrate control regimes. The four colored lines indicate regime transitions for the four typical scenarios in Fig. 1, when SO_4_^2−^ is varied by ±70% or when the free ammonia or free nitrate for partitioning is exhausted (see Supplementary Information S3). Shaded area indicate the regions insensitive or susceptible to *S*_f_ variations at the range of 0 ∼5, which is the corresponding *S*_f_ ranges of the four representative scenarios.

Unlike NH_3_^T^ and HNO_3_^T^, which are both positively correlated with NO_3_^−^, a decrease in sulfate enhances NO_3_^−^ formation since the *S*(SO_4_^2−^) is always negative (Equation 6). This influence is usually more pronounced at lower p*K*_AN_ levels and when *A*_ex_ < 1 (Fig. 3a, b, dark-blue areas), such as the NE-US and YRD scenarios. Under such conditions, NH_3_ is the limiting species, a decrease in sulfate increases NH_3_ availability, thereby increasing NO_3_^−^. Notably, a net particulate matter (PM) mass increase zone may occur when *S*(SO_4_^2−^) <−1.55, where 1.55 is the ratio of molar mass of SO_4_^2−^ to NO_3_^−^. In this zone, a one-mole decrease in SO_4_^2−^ would result in 1.55-mole increase in NO_3_^−^, resulting in a net PM increase. Nevertheless, such conditions are rare given the generally NH₃-excess environment, and in nearly all urban areas sulfate reductions contribute to decreasing PM_2.5_ and improving air quality. In comparison, when NH_3_ is in great excess (*A*_ex_ >>1, e.g. the NCP scenario), free NH_3_ released by reducing sulfate has only a minor effect on NO_3_^−^. Similarly, at higher p*K*_AN_ levels when meteorology takes control, the nitrate is nearly insensitive to any emissions given the predominant gas-phase partitioning (e.g. SE-US scenario, Fig. 1d).

In general, the sensitivity of nitrate to sulfate decreases as sulfate levels decline (diminishing marginal effect). When *S*_f_ decreases, the net PM increase zone at *A*_ex_ < 1 condition expands to higher p*K*_AN_ levels (Fig. 3a, b). When NH_3_ is in excess (e.g. *A*_ex_ = 2), both the peak |*S*(SO_4_^2−^)| values and the corresponding p*K*_AN_ levels are negatively correlated with *S*_f_ levels (Fig. 3c), resulting in shrinkage of sulfate-sensitive zones. Higher |*S*(SO_4_^2−^)| typically occurs under moderate p*K*_AN_ levels, such as the clean winter (e.g. 270 K with *L*_w_ ∼1 ug m^−3^) or polluted summer (e.g. 300 K with *L*_w_ ∼10 ug m^−3^) conditions (Fig. 2a).

Sulfate not only influences the nitrate concentration, but also the nitrate control regimes. Other conditions kept the same, decreasing sulfate would increase both *A*_ex_ and p*K*_AN_, pushing the situation toward the upper-right corner of Fig. 2. The *A*_ex_ increase is usually the dominant effect. Correspondingly, decreasing sulfate pushes the regime from NH_3_^T^ toward Trans and then to HNO_3_^T^, as demonstrated by the pathways for the four representative scenarios (four lines in Fig. 3d). This effect has contributed to the regime transition of China from 2013 to 2019 (Fig. S5a). Assuming other conditions are the same as in 2019, if the sulfate is still at the 2013 level, then most regions of YRD and some regions of Central China would reside in the NH_3_^T^ regime in winter, while in other seasons, the occurrence of NH_3_^T^ regime would also be much higher.

The decreasing *S*_f_ also reduces the occurrence of the meteorology-limited regime (as indicated by the areas it covers) while increasing the possibility of other regimes (Fig. 3d, changes of the shaded area). Over continental urban areas, *S*_f_ averaged 3.2 and can sometimes reach over 6 (Fig. S5b). Therefore, the meteorology-limited regime can be found at p*K*_AN_ of ∼0, more readily than in the *S*_f_ = 0 case (Fig. S5c). Further reductions in sulfate would increase the sensitivity of nitrate to NH_3_^T^ or HNO_3_^T^.

Non-volatile cations, mainly crustal ions like Na^+^, Ca^2+^, K^+^, and Mg^2+^, act in the opposite manner to sulfate. If changes in climate, land use, or emissions were to substantially increase dust loading and the subsequent contribution of non-volatile cations, then this would act approximately equivalently to decreasing sulfate. That is, increasing *A*_ex_ and thus pushing the nitrate control regime toward the HNO_3_^T^ regime. Consquently, the nitrate sensitivity to HNO_3_^T^ or NH_3_^T^ would increase, especially at moderate p*K*_AN_ levels.

### Global distributions

**Figure 4.**
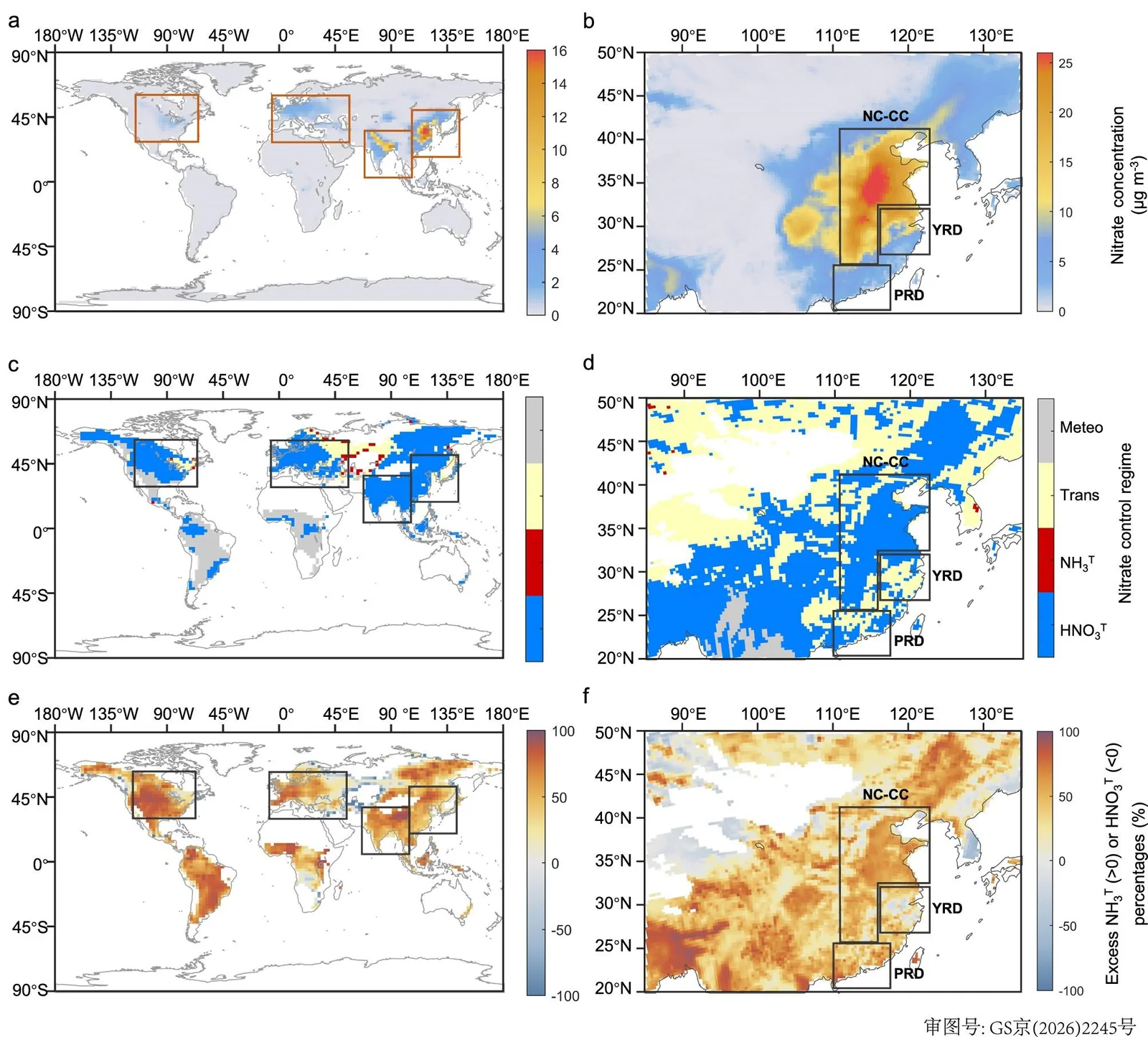
Spatial distribution of nitrate and its control regimes over continental surface regions in 2019. (a, c, e) Global simulations by GEOS-Chem, and (b, d, f) regional simulations focusing middle and eastern part of the Chinese mainland by WRF-CMAQ, for (a, b) annual average nitrate concentrations, (c, d) nitrate control regimes, and (e, f) the excess NH_3_^T^ and HNO_3_^T^ percentages. The regimes shown in panels (c, d) are determined based on Equations 4–5 with GEOS-Chem and WRF-CMAQ modeled concentrations and meteorology. In panels (e, f), positive values and negative values correspond to conditions when NH_3_^T^ and HNO_3_^T^ are in excess, respectively. The squares in panels (a, c, e) show the regions with relative high nitrate concentrations, which are middle and eastern parts of China, India, Europe, and northern USA. The squares in panels (b, d, f) indicate the approximate locations of NC-CC, YRD, and PRD, respectively.

Figure 4 shows the simulated distribution of nitrate concentration and its control regimes globally and in China in 2019 (see Methods M3 and Supplemetary Information S4). Here, the control regimes in Fig. 4c and d are determined based on Equations 4 and 5 with GEOS-Chem and WRF-CMAQ modeled concentrations and meteorology, respectively. As indicated by squares in Fig. 4a, regions with elevated nitrate concentrations mainly include the middle and eastern parts of China, India, Europe, and the northern part of USA. All these regions lie largely in the HNO_3_^T^ regime (Fig. 4c), highlighting the importance of HNO_3_^T^-control over the regions with typical nitrate pollution. Moreover, ∼50% NH_3_ excess typically exists in these regions (Fig. 4e), indicating that ∼50% of NH_3_ would need to be reduced first before ammonia control is effective. The NH_3_^T^ regime clustered around central Asia and Russia, which is due to the low anthropogenic NH_3_ emissions in the Community Emissions Data System v2 inventory [44]. In comparison, the southernmost part of the United States, most of Latin America and Africa lie in the meteorology-limited regime, due to the high temperature and/or relatively low PM loads. Correspondingly, the nitrate concentrations over these regions are low. The occurrence of the NH_3_^T^ regime is rare, consistent with previous findings that the atmosphere is generally alkaline nowadays [37,45,46].

We further focus on China, which is a global ‘hot spot’ of nitrate (Fig. 4a). Similarly, regions with the highest nitrate concentrations, that is, the North China Plain and Central China (NC-CC) region, nearly all lie in the HNO_3_^T^ regime, and ∼40% of NH_3_ must first be reduced before NH_3_ control becomes comparably effective as HNO_3_^T^ control (Fig. 4b, d and f). While transition regimes exist in the YRD and Pearl River Delta (PRD) region, the *S*(HNO_3_^T^) to *S*(NH_3_^T^) ratio is typically much larger than 1 (Fig. S6a, b). These conclusions are further supported by long-term observations in Shanghai (Fig. S6c, d). The seasonal variation in the control regime distributions generally agrees with the annual average, while in summer, more southern regions lie in the meteorology-limited regime due to the higher temperature and cleaner conditions, and in winter, the NH_3_ control can be more effective at some locations in the YRD region (Fig. S5a). Overall, nitrate is much more sensitive to HNO_3_^T^ than to NH_3_^T^ in China, in good agreement with the GEOS-Chem simulations.

We also note that *S*(HNO_3_^T^) cannot be directly translated into the nitrate sensitivity to NOx emissions *S*(NOx). Rather, their relationship can be roughly represented as


(8a) \begin{eqnarray*}
{\mathit{S}}\left( {{\mathrm{NOx}}} \right) = {\eta }_{{\mathrm{NOx}}}\cdot{\mathit{S}}\left( {{\mathrm{HN}}{{\mathrm{O}}}_3^{\mathrm{\,\,T}}} \right),
\end{eqnarray*}

where η_NOx_ is the NOx-to-HNO_3_ conversion ratio that varies with atmospheric chemistry and transport, etc. Moreover, as NH_3_^T^ is less susceptible to chemical conversions, Equation 8 provides a reference threshold of NOx-to-HNO_3_ chemical production ratio of


(8b) \begin{eqnarray*}
{\eta }_{{\mathrm{NOx}},{\mathrm{thr}}} = {\mathit{S}}\left( {{\mathrm{N}}{{\mathrm{H}}}_3^{\mathrm{\,\,T}}} \right)/{\mathit{S}}\left( {{\mathrm{HN}}{{\mathrm{O}}}_3^{\mathrm{\,\,T}}} \right)
\end{eqnarray*}

at which point the *S*(NOx) would equal *S*(NH_3_^T^). In other words, the NOx control would be more effective than NH_3_ only when η_NOx_ is greater than η_NOx, thr_. As a first-order estimate, we adopted the nitrogen oxidation ratio (the molar ratio of [HNO_3_^T^] to [HNO_3_^T^] + [NOx]) as the proxy of η_NOx_. The results show that the nitrate control regimes remain largely unchanged given the large NH_3_ excess and therefore the small η_NOx,thr_ (see more discussions in Supplementary Information S5 and Fig. S7). More investigations on η_NOx_ and the influencing factors are encouraged in future work.

### Comparison with previous studies

While our study suggests the dominant presence of HNO_3_^T^ regime in China, some previous studies drew the opposite conclusion [2,16,17]. These apparent discrepancies can be largely reconciled by differences in sensitivity definitions, uncertainties in simulated ammonia excess, and the efficiency metric considered, as detailed in Supplementary Information S6 and Figs S8 and S9. For instance, our sensitivity is defined on an absolute molar basis, whereas some previous studies used percentage responses [2,16,17]. As NH_3_^T^ is often 5–10 times more abundant than HNO_3_^T^ over ammonium-nitrate-dominated regions, a 1% NH_3_^T^ reduction represent a much larger molar perturbation than a 1% HNO_3_^T^ reduction. Moreover, the inferred regime can be highly sensitive to the simulated extent of ammonia excess under low p*K*_AN_ conditions and when *A*_ex_ is around 1 (Fig. 2), such as the polluted periods in winter NCP or YRD. Under these conditions, small shifts due to modeling uncertainties, etc., can rapidly move the system from the NH_3_^T^-control to the HNO_3_^T^ regime (Fig. 2f). Moreover, this initial difference may be further amplified in atmospheric Chemical Transport Models (CTM) through the lifetime effect as discussed below.

Beyond the direct effect on local nitrate concentrations, gas-particle partitioning also influences total nitrate atmospheric lifetime via dry deposition. Particle-phase nitrate exhibits much lower dry-deposition velocities than gaseous HNO_3_ [2,5]. Therefore, enhanced particle-phase partitioning retards nitrate removal, extending its lifetime and favoring accumulation, which has been suggested as one major contributor to the formation of nitrate pollution [2,5]. This lifetime effect can amplify small initial thermodynamic sensitivity discrepancies during transport, which is especially relevant near regime boundaries (e.g. the winter NCP or YRD scenario as discussed above). Therefore, accurate representation of NH_3_ emissions and availability is particularly important under such conditions.

Our analytical framework cannot replace full atmospheric CTM simulations, but provides a mechanistic interpretation of their results. Full CTM simulations remain essential as they explicitly represent emissions, chemical production, transport, meteorology, depositions, and other coupled feedbacks. In addition, they provide the base-scenario spatial distributions required to diagnose nitrate control regimes. Further, they are indispensable for translating secondary product sensitivities to emitted precursors (e.g. *S*(HNO_3_^T^) into *S*(NOx), or *S*(SO_4_^2−^) into *S*(SO_2_)). In turn, using those modeled concentrations and meteorological fields, our framework can partly simplify the sensitivity analysis that would otherwise require many perturbation simulations, and can help separate the contributions of different processes to the apparent model sensitivity. In particular, by recasting nitrate partitioning into the *A*_ex_–p*K*_AN_ coordinates, this framework helps identify the key factors for accurate nitrate simulations, that is, ammonia excess (*A*_ex_) and the ammonium-nitrate activity coefficient (γ_AN_). In comparison, accurate simulation of aerosol pH may play a minor role considering its counteracting effect on ammonia and nitrate partitioning. Thus, this framework functions as an interpretive layer for CTM models, not a substitute.

Importantly, we note that chemical effectiveness does not equate to cost-effectiveness, that is, the marginal abatement costs for NH_3_ or NOx may differ significantly by region [16,17]. Correspondingly, practical policy effectiveness requires integrated consideration of emission sector structure, oxidation chemistry, and regional economics, which warrants investigation in future studies. For these purposes, the derived nitrate sensitivities here (Equations 4–7) can provide the reference threshold of thermodynamic partitioning processes, which can help simplify estimations of other efficiencies, following the procedures as shown in the discussion of NOx-to-HNO_3_ chemical production (Equation 8; see more discussions in Supplementary Information S5–S6).

### Implications

The nitrate control regime classifications proposed here provide an important scientific basis and practical quantitative criteria for air pollution controls. While previous sensitivity studies have suggested that nitrate tends to be more sensitive to HNO_3_^T^ under NH_3_-excess conditions (e.g. Fig. 1e–h), our framework advances previous understanding in three respects. First, it provided a theoretical foundation to generalize this pattern confidently. Second, it yields an analytically derived quantitative threshold separating NH_3_-excess and NH_3_-deficient conditions, thereby unifying the diverse indicators proposed previously [2,19,46–48]. Third, it demonstrated that *A*_ex_ alone does not govern nitrate sensitivity. Even at constant NH_3_-excess levels, sensitivities can vary much with p*K*_AN_, explaining the diverse response patterns in Fig. 1e–h. This *A*_ex_–p*K*_AN_ framework, which simply requires inputs of widely accessible major inorganic chemical profiles and meteorological conditions of RH and temperature (Method M2), thereby enables direct comparisons of multiple scenarios in one plot, instead of case-by-case illustrations (Fig. 1e–h). Additionally, this framework identifies a previously unrecognized meteorology-limited regime.

Our results suggest that reducing HNO_3_^T^, and by extension NOx, is more effective than NH_3_ control for areas with typical nitrate pollution like China, considering the great excess of ammonia. Conversely, sulfate reductions increase both nitrate concentrations and its sensitivities to HNO_3_^T^ and NH_3_^T^. Although *S*(SO_4_^2−^) translates non-linearly to *S*(SO_2_) due to complex conversion chemistry, the general trend would hold only if SO_2_ and sulfate correlate positively.

The applicability of this framework requires two prerequisites, that nitrate predominantly exists as ammonium nitrate, and that gas-particle equilibrium is a reasonable approximation (Method M2). Therefore, it should not be overextended to conditions where chloride and non-volatile cations dominate, or to cases with strong kinetic limitations, such as the coarse-mode sea salt and dust particles, or low-RH conditions (RH < ∼40%) where viscous organic aerosols govern the kinetics [1,49,50].

The multiphase dissociation equilibrium of ammonium nitrate reveals how the meteorological conditions and emission profiles jointly govern nitrate partitioning, the implications of which go beyond air pollution. It can be readily applied in studies of nitrogen deposition [5,8], acid rain formation [16,51], aerosol condensational growth [4,52], the climate effects of reactive nitrogen [3], as well as the environmental impact assessment of ammonia as a zero-carbon fuel and energy carrier [53]. Our results highlight the need for continued NOx control in nitrate-dominated areas, and reveal the complex interactions among human activities, meteorology, and atmospheric multiphase processes.

## MATERIALS AND METHODS

### M1. Long-term observation dataset

Multiple observation datasets were used to investigate the nitrate sensitivity regimes and the typical ranges of several key parameters. The scenarios as shown in Fig. 1 and Fig. S8 are based on average conditions in summer SE-US [31,32], winter NE-US [32,33], Shanghai in fall (based on our own measurement), and winter Beijing [34], respectively, as detailed in Table S1. In Fig. 2a, Figs S3, S5 and S6, long-term datasets with hourly to daily resolutions are adopted to show the variation ranges. These include observations conducted at SE-US from 2008 to 2016 [35–37], six sites of Canada (CA) from 2007 to 2016 [38], urban India (India) from 2017 to 2018 [39], NCP in 2013 [40,41], and YRD during 2013 and 2019 [42]. See details in Table S3.

### M2. Derivation of multiphase dissociation equilibrium of ammonium nitrate

#### Relationship with the multiphase buffer theory

The multiphase dissociation equilibrium can be derived from the multiphase buffer theory, which quantitatively explained how the acidity, gas-particle partitioning, and acid dissociation equilibrium are inter-correlated for volatile acids and bases in a multiphase aerosol system. Applying this theory to the multiphase NH_4_^+^/NH_3_ and HNO_3_/NO_3_^−^ systems, respectively, we have


(M1) \begin{eqnarray*}
K{}_{{\mathrm{a,NH3}}}^{*,ni} &=& \frac{{{\mathrm{[}}{{\mathrm{H}}}^{\mathrm{ + }}( {{\mathrm{aq}}} ){\mathrm{][N}}{{\mathrm{H}}}_{\mathrm{3}}{\mathrm{(g)]}}}}{{{\mathrm{[N}}{{\mathrm{H}}}_4^{\mathrm{\,\, + }}( {{\mathrm{aq}}} ){\mathrm{]}}}} \\
&=& \frac{{{\gamma }_{{\mathrm{NH4 + }}}}}{{{\gamma }_{{\mathrm{H + }}}}}{K}_{{\mathrm{a,NH3}}}\frac{{{\rho }_w}}{{{H}_{{\mathrm{NH3}}}\ R\ T\ {L}_{\mathrm{w}}}},
\end{eqnarray*}

(M2) \begin{eqnarray*}
K{}_{{\mathrm{a,HNO3}}}^{*,ni} &=& \frac{{{\mathrm{[}}{{\mathrm{H}}}^{\mathrm{ + }}( {{\mathrm{aq}}} ){\mathrm{][N}}{{\mathrm{O}}}_{\mathrm{3}}^{\mathrm{\,\, - }}( {{\mathrm{aq}}}){\mathrm{]}}}}{{{\mathrm{[HN}}{{\mathrm{O}}}_{\mathrm{3}}{\mathrm{(g)]}}}}\\
&=& {K}_{{\mathrm{a,HNO3}}}\frac{{{H}_{{\mathrm{HNO3}}}\ R\ T\ {L}_{\mathrm{w}}}}{{{\gamma }_{{\mathrm{H + }}}{\gamma }_{{\mathrm{NO3 - }}}{\rho }_w}},
\end{eqnarray*}

where the symbol definitions are the same as in Equation 1. Dividing Equation M2 by M1, we can eliminate the influence of aerosol acidity (i.e. [H^+^]), and get Equation 1b in the main text, which represents the overall relationship of acid dissociation equilibrium and gas-particle partitioning for the HNO_3_ and NH_3_ systems. Further combining constraints from mass conservation and liquid phase charge balance, we can derive the basic equilibirum equation (Equation 2a) in the main text. See detailed derivation processes in Supplementary Text S3.

#### Required inputs

The required inputs to solve the multiphase dissociation equilibrium are the same as most thermodynamic models, which include: (i) the total (gas + particle) concentration of major semi-volatile inorganic species, including HNO_3_^T^, NH_3_^T^, and HCl^T^; (ii) the particle-phase concentration of major non-volatile inorganic species, including SO_4_^2−^, Na^+^, Ca^2+^, K^+^, and Mg^2+^; and (iii) meteorological parameters of RH and temperature.

#### Constraints and applicability

The basic equation for multiphase dissociation equilibrium (Equation 2a) can be adopted to predict particulate nitrates only when the following criteria are satisfied. (i) Negligible influence of Cl^−^ (<10% of total anions); (ii) equivalent charge of NVCs is smaller than that of total anions; and (iii) equivalent charge of non-volatile anions (i.e. sulfate) is smaller than that of total cations. In addition, to enable the achievement of gas-particle equilibrium, we confined the application of this framework to fine-mode particles under RH over 40% only [1,49,50]. See detailed expression of the above criteria in Supplementary Text S3.

### M3. Model simulations

Data in Fig. 4a, c and e are from the 3-D global chemical transport model GEOS-Chem v.14.2.3 (https://zenodo.org/records/10246625; last access: 2025/4/1) for 2019, at a spatial resolution to 2.0° latitude × 2.5° longitude with 47 vertical layers [54]. Data shown in Fig. 4b, d and f were results of WRF-CMAQ simulations over China in 2013 and 2019, with detailed model configurations and validations described elsewhere [55]. See more simulation details in Supplementary Text S4.

## Supplementary Material

nwag481_Supplemental_File

## Data Availability

All data are available in the main text or the supplementary materials. The GEOS-Chem model code used in this study is permanently archived in Zenodo (29).

## References

[1] Seinfeld JH, Pandis SN. Atmospheric Chemistry and Physics: from Air Pollution to Climate Change. Hoboken: John Wiley & Sons, 2016.

[2] Zhai S, Jacob DJ, Wang X et al. Control of particulate nitrate air pollution in China. Nat Geosci 2021; 14: 389–95. 10.1038/s41561-021-00726-z

[3] Gong C, Tian H, Liao H et al. Global net climate effects of anthropogenic reactive nitrogen. Nature 2024; 632: 557–63. 10.1038/s41586-024-07714-4 39048828 PMC11324526

[4] Wang M, Kong W, Marten R et al. Rapid growth of new atmospheric particles by nitric acid and ammonia condensation. Nature 2020; 581: 184–9. 10.1038/s41586-020-2270-4 32405020 PMC7334196

[5] Nenes A, Pandis SN, Kanakidou M et al. Aerosol acidity and liquid water content regulate the dry deposition of inorganic reactive nitrogen. Atmos Chem Phys 2021; 21: 6023–33. 10.5194/acp-21-6023-2021

[6] Xu L, Penner JE. Global simulations of nitrate and ammonium aerosols and their radiative effects. Atmos Chem Phys 2012; 12: 9479–504. 10.5194/acp-12-9479-2012

[7] Makkonen R, Romakkaniemi S, Kokkola H et al. Brightening of the global cloud field by nitric acid and the associated radiative forcing. Atmos Chem Phys 2012; 12: 7625–33. 10.5194/acp-12-7625-2012

[8] Liu M, Shang F, Lu X et al. Unexpected response of nitrogen deposition to nitrogen oxide controls and implications for land carbon sink. Nat Commun 2022; 13: 3126. 10.1038/s41467-022-30854-y 35668096 PMC9170707

[9] Li HY, Cheng J, Zhang Q et al. Rapid transition in winter aerosol composition in Beijing from 2014 to 2017: response to clean air actions. Atmos Chem Phys 2019; 19: 11485–99. 10.5194/acp-19-11485-2019

[10] Wang J, Gao J, Che F et al. Decade-long trends in chemical component properties of PM2.5 in Beijing, China (2011–2020). Sci Total Environ 2022; 832: 154664. 10.1016/j.scitotenv.2022.154664 35314233

[11] Qi L, Zheng H, Ding D et al. Responses of sulfate and nitrate to anthropogenic emission changes in eastern China—in perspective of long-term variations. Sci Total Environ 2023; 855: 158875. 10.1016/j.scitotenv.2022.158875 36126708

[12] Wen L, Chen J, Yang L et al. Enhanced formation of fine particulate nitrate at a rural site on the North China Plain in summer: the important roles of ammonia and ozone. Atmos Environ 2015; 101: 294–302. 10.1016/j.atmosenv.2014.11.037

[13] Wen Z, Xu W, Pan XY et al. Effects of reactive nitrogen gases on the aerosol formation in Beijing from late autumn to early spring. Environ Res Lett 2021; 16: 025005. 10.1088/1748-9326/abd973

[14] Tian X, Yu H, Wei Y et al. Gas-particle partitioning process contributes more to nitrate dominated air pollution than oxidation process in northern China. Aerosol Sci Technol 2024; 58: 181–94. 10.1080/02786826.2023.2294944

[15] Li H, Duan F, Ma Y et al. Case study of spring haze in Beijing: characteristics, formation processes, secondary transition, and regional transportation. Environ Pollut 2018; 242: 544–54. 10.1016/j.envpol.2018.07.001 30007265

[16] Liu M, Huang X, Song Y et al. Ammonia emission control in China would mitigate haze pollution and nitrogen deposition, but worsen acid rain. Proc Natl Acad Sci USA 2019; 116: 7760–5. 10.1073/pnas.1814880116 30936298 PMC6475379

[17] Gu B, Zhang L, Van Dingenen R et al. Abating ammonia is more cost-effective than nitrogen oxides for mitigating PM_2.5_air pollution. Science 2021; 374: 758–62. 10.1126/science.abf8623 34735244

[18] Wang Y, Zhang QQ, He K et al. Sulfate-nitrate-ammonium aerosols over China: response to 2000–2015 emission changes of sulfur dioxide, nitrogen oxides, and ammonia. Atmos Chem Phys 2013; 13: 2635–52. 10.5194/acp-13-2635-2013

[19] Xu ZY, Liu MX, Zhang MS et al. High efficiency of livestock ammonia emission controls in alleviating particulate nitrate during a severe winter haze episode in northern China. Atmos Chem Phys 2019; 19: 5605–13. 10.5194/acp-19-5605-2019

[20] Guo H, Otjes R, Schlag P et al. Effectiveness of ammonia reduction on control of fine particle nitrate. Atmos Chem Phys 2018; 18: 12241–56. 10.5194/acp-18-12241-2018

[21] Zang H, Zhao Y, Huo JT et al. High atmospheric oxidation capacity drives wintertime nitrate pollution in the eastern Yangtze River Delta of China. Atmos Chem Phys 2022; 22: 4355–74. 10.5194/acp-22-4355-2022

[22] Wei Y, Nenes A, Gao J et al. Abundant nitrogen oxide and weakly acidic environment synergistically promote daytime particulate nitrate pollution. J Hazard Mater 2023; 456: 131655. 10.1016/j.jhazmat.2023.131655 37216807

[23] Wen L, Xue L, Dong C et al. Reduced atmospheric sulfate enhances fine particulate nitrate formation in eastern China. Sci Total Environ 2023; 898: 165303. 10.1016/j.scitotenv.2023.165303 37419351

[24] Shi X, Nenes A, Xiao Z et al. High-resolution data sets unravel the effects of sources and meteorological conditions on nitrate and its gas-particle partitioning. Environ Sci Technol 2019; 53: 3048–57. 10.1021/acs.est.8b06524 30793889

[25] Vasilakos P, Russell A, Weber R et al. Understanding nitrate formation in a world with less sulfate. Atmos Chem Phys 2018; 18: 12765–75. 10.5194/acp-18-12765-2018

[26] Duan J, Huang RJ, Li YJ et al. Summertime and wintertime atmospheric processes of secondary aerosol in Beijing. Atmos Chem Phys 2020; 20: 3793–807. 10.5194/acp-20-3793-2020

[27] Li Y, Lei L, Sun J et al. Significant reductions in secondary aerosols after the three-year action plan in Beijing summer. Environ Sci Technol 2023; 57: 15945–55. 10.1021/acs.est.3c02417 37823561

[28] Nenes A, Pandis SN, Weber RJ et al. Aerosol pH and liquid water content determine when particulate matter is sensitive to ammonia and nitrate availability. Atmos Chem Phys 2020; 20: 3249–58. 10.5194/acp-20-3249-2020

[29] Zheng G, Su H, Wang S et al. Multiphase buffer theory explains contrasts in atmospheric aerosol acidity. Science 2020; 369: 1374–7. 10.1126/science.aba3719 32913103

[30] Fountoukis C, Nenes A. ISORROPIA II: a computationally efficient thermodynamic equilibrium model for K^+^-Ca^2+^-Mg^2+^-NH_4_^+^-Na^+^-SO_4_^2−^-NO_3_^−^-Cl^−^-H_2_O aerosols. Atmos Chem Phys 2007; 7: 4639–59. 10.5194/acp-7-4639-2007

[31] Guo H, Xu L, Bougiatioti A et al. Fine-particle water and pH in the southeastern United States. Atmos Chem Phys 2015; 15: 5211. 10.5194/acp-15-5211-2015

[32] Guo H, Weber RJ, Nenes A. High levels of ammonia do not raise fine particle pH sufficiently to yield nitrogen oxide-dominated sulfate production. Sci Rep 2017; 7: 12109. 10.1038/s41598-017-11704-0 28935864 PMC5608889

[33] Guo H, Sullivan AP, Campuzano-Jost P et al. Fine particle pH and the partitioning of nitric acid during winter in the northeastern United States. J Geophys Res: Atmos 2016; 121: 10355–76. 10.1002/2016JD025311

[34] Ding J, Zhao PS, Su J et al. Aerosol pH and its driving factors in Beijing. Atmos Chem Phys 2019; 19: 7939–54. 10.5194/acp-19-7939-2019

[35] Edgerton ES, Hartsell BE, Saylor RD et al. The Southeastern Aerosol Research and Characterization Study, part 3: continuous measurements of fine particulate matter mass and composition. J Air Waste Manage Assoc 2006; 56: 1325–41. 10.1080/10473289.2006.10464585 17004687

[36] Weber RJ, Guo H, Russell AG et al. High aerosol acidity despite declining atmospheric sulfate concentrations over the past 15 years. Letter. Nature Geosci 2016; 9: 282–5. 10.1038/ngeo2665

[37] Zheng G, Su H, Cheng Y. Revisiting the key driving processes of the decadal trend of aerosol acidity in the U.S. ACS Environ Au 2022; 2: 346–53. 10.1021/acsenvironau.1c00055 37101965 PMC10125332

[38] Tao Y, Murphy JG. The sensitivity of PM2.5 acidity to meteorological parameters and chemical composition changes: 10-year records from six Canadian monitoring sites. Atmos Chem Phys 2019; 19: 9309–20. 10.5194/acp-19-9309-2019

[39] Chen Y, Wang Y, Nenes A et al. Ammonium chloride associated aerosol liquid water enhances haze in Delhi, India. Environ Sci Technol 2022; 56: 7163–73. 10.1021/acs.est.2c00650 35483018 PMC9178790

[40] Zheng GJ, Duan FK, Su H et al. Exploring the severe winter haze in Beijing: the impact of synoptic weather, regional transport and heterogeneous reactions. Atmos Chem Phys 2015; 15: 2969–83. 10.5194/acp-15-2969-2015

[41] Cheng Y, Zheng G, Wei C et al. Reactive nitrogen chemistry in aerosol water as a source of sulfate during haze events in China. Sci Adv 2016; 2: e1601530. 10.1126/sciadv.1601530 28028539 PMC5176349

[42] Zhou M, Zheng G, Wang H et al. Long-term trends and drivers of aerosol pH in eastern China. Atmos Chem Phys 2022; 22: 13833–44. 10.5194/acp-22-13833-2022

[43] Zhao Q, Nenes A, Yu H et al. Using high-temporal-resolution ambient data to investigate gas-particle partitioning of ammonium over different seasons. Environ Sci Technol 2020; 54: 9834–43. 10.1021/acs.est.9b07302 32677824

[44] McDuffie EE, Smith SJ, O’Rourke P et al. A global anthropogenic emission inventory of atmospheric pollutants from sector-and fuel-specific sources (1970–2017): an application of the Community Emissions Data System (CEDS). Earth Syst Sci Data 2020; 12: 3413–42. 10.5194/essd-12-3413-2020

[45] Karydis VA, Tsimpidi AP, Pozzer A et al. How alkaline compounds control atmospheric aerosol particle acidity. Atmos Chem Phys 2021; 21: 14983–5001. 10.5194/acp-21-14983-2021

[46] Zheng G, Su H, Wan R et al. Rising alkali-to-acid ratios in the atmosphere may correspond to increased aerosol acidity. Environ Sci Technol 2024; 58: 16517–24. 10.1021/acs.est.4c06860 39231580

[47] Ansari AS, Pandis SN. Response of inorganic PM to precursor concentrations. Environ Sci Technol 1998; 32: 2706–14. 10.1021/es971130j

[48] Pinder RW, Dennis RL, Bhave PV. Observable indicators of the sensitivity of PM2.5 nitrate to emission reductions—Part I: derivation of the adjusted gas ratio and applicability at regulatory-relevant time scales. Atmos Environ 2008; 42: 1275–86. 10.1016/j.atmosenv.2007.10.039

[49] Fountoukis C, Nenes A, Sullivan A et al. Thermodynamic characterization of Mexico City aerosol during MILAGRO 2006. Atmos Chem Phys 2009; 9: 2141–56. 10.5194/acp-9-2141-2009

[50] Preston TC, Zuend A. Equilibration times in viscous and viscoelastic aerosol particles. Environ Sci-Atmos 2022; 2: 1376–88. 10.1039/D2EA00065B

[51] Zheng G, Su H, Cheng Y. Role of carbon dioxide, ammonia, and organic acids in buffering atmospheric acidity: the distinct contribution in clouds and aerosols. Environ Sci Technol 2023; 57: 12571–82. 10.1021/acs.est.2c09851 37599651 PMC10469486

[52] Wang M, Xiao M, Bertozzi B et al. Synergistic HNO_3_–H_2_SO_4_–NH_3_ upper tropospheric particle formation. Nature 2022; 605: 483–9. 10.1038/s41586-022-04605-4 35585346 PMC9117139

[53] Ma N, Zhao WH, Wang WZ et al. Large scale of green hydrogen storage: opportunities and challenges. Int J Hydrogen Energ 2024; 50: 379–96. 10.1016/j.ijhydene.2023.09.021

[54] Bey I, Jacob DJ, Yantosca RM et al. Global modeling of tropospheric chemistry with assimilated meteorology: model description and evaluation. J Geophys Res: Atmos 2001; 106: 23073–95. 10.1029/2001JD000807

[55] Geng G, Liu Y, Liu Y et al. Efficacy of China’s clean air actions to tackle PM2.5 pollution between 2013 and 2020. Nat Geosci 2024; 17: 987–94. 10.1038/s41561-024-01540-z

