# Supplementary material for: Control regimes of nitrate air pollution reconciled by multiphase dissociation equilibrium
Source: Natl Sci Rev. 2026 Aug 11;13(17):nwag481. doi: 10.1093/nsr/nwag481 (PMC13556601; doi:10.1093/nsr/nwag481)
Supplement: Supplementary file 1 [file nwag481_supplemental_file.pdf]

Supplementary Materials for

Control regimes of nitrate air pollution reconciled by multiphase dissociation equilibrium

Guangjie Zheng* *et al.*

Corresponding author: zgj123@tsinghua.edu.cn

The PDF file includes:

Supplementary Text

Figs. S1 to S9

Tables S1 to S3

References

Supplementary Text

S1. Previously proposed dominant influencing factors of nitrate formation

Previous studies have intensively investigated into the complex influencing factors of nitrate formation. First, as nitrate is a secondary species, some studies emphasized the fundamental strategy for nitrate control is to control its precursor NO_x, or to limit the efficiency of NO_x-HNO_3 conversions through modulating the atmospheric oxidizing capacity [10,20-22,49-59] (Fig. S1, processes in the dashed rectangles to the right). In comparison, as nitrate is volatile, some other studies considered that it's more effective to control the nitrate gas-particle partitioning, as indicated by the particle-phase fraction $f_{p,\ NO3}$. The $f_{p,\ NO3}$, however, is further influenced by the atmospheric chemical profiles and meteorological conditions through complex processes (Fig. S1, left part), and its dominant controlling factors remain highly debated among studies [18,23-25]. Some studies [19] found that the sensitivity of nitrate to HNO_3 and NH_3 is highly susceptible to ambient NH_3 availability based on the ISORROPIA simulations, but the theoretical explanation and the applicability under different scenarios are not given. It was also pointed out that due to the significant impact of meteorological conditions on nitrate formation, the relative dominance between NOx conversion versus gas-particle partitioning can exhibit significant seasonal or even diurnal variations[11,12,26,27].

Recently, some studies [5,20,24,41,42,60] proposed that the sensitivity of nitrate to nitric acid versus ammonia can be determined by the aerosol water content L_w and aerosol acidity (or pH). However, its application suffers from several major drawbacks. First, the detailed regime classification in the L_w – pH plots vary significantly with temperature, and ambient data with different temperatures cannot be plotted on the same L_w – pH plot to be compared directly. Second, aerosol pH and L_w are not independent variables; rather, aerosol pH is largely regulated by L_w [43]. Therefore, this method cannot fully decouple the overall influence of L_w. Moreover, aerosol pH depends further on a series of complex influencing factors, including both meteorological conditions and chemical profiles (Fig. S1, left part). In fact, the response of aerosol pH itself to different emissions is still an unresolved issue under intensive investigation [44,45,61]. Therefore, choosing pH as one major axis does not help simplify the picture of dominant factors in nitrate partitioning. Overall, one cannot decouple the influence of meteorology versus chemical profiles, or understand the nitrate response to the emissions of different species with this method.

S2. Major uncertainty sources of pK_{AN} and the influence on regime classifications

Based on Eq. 1, we see that the uncertainty of pK_{AN} at given meteorological conditions comes from two major parameters, i.e. the activity coefficient of ammonium nitrate γ_{AN}, and the aerosol water content L_w. The corresponding pK_{AN} uncertainties may further influence the regime classifications. Here these issues were examined with the ISORROPIA model (version 2.3)[40], based on the observational datasets as described in Method M1, and the daily WRF-CMAQ and GEOS-Chem simulations over continental areas within the ammonium-nitrate multiphase dissociation regimes as detailed in Method M3. Note that we focused on scenarios when the proposed framework applies, i.e. when conditions satisfy the criterion as outlined in Method M2.

S2.1 Variation ranges of activity coefficient γ_{AN}

For all the investigated datasets, γ^2_{AN} typically spans from 0.01 to 0.15, with model outputs predominantly clustering around 0.05 (Fig. S3A). As pK_{AN} is proportional to log(γ^2_{AN}) (Eq. 1b), this γ^2_{AN} variation range corresponds to a pK_{AN} fluctuation of ~ 1 unit. More investigations on the influencing factors of γ^2_{AN} are provided in our recent study [62].

S2.2 Variations of aerosol water content L_w

The L_w depends on relative humidity RH, aerosol mass concentrations and the mass-based hygroscopicity. To quantify the mass-based hygroscopicity, we introduce the specific water content q_w, which is defined as the ratio of L_w to the dry mass of hygroscopic species. For a given site where the emissions are relatively stable, variations in total aerosol mass concentration are largely regulated by meteorological conditions. In comparison, the q_w depends mainly on the chemical profiles, especially for Accumulation mode particles which have larger particle sizes and are the major mass concentration contributors. Correspondingly, particle-phase chemical compositions influence L_w mainly through q_w.

As inorganic species are usually the major contributors of L_w [30], we first investigated the q_w of inorganic species, namely the ratio of L_w to the total dry mass of inorganic species (sulfate, nitrate, ammonium and NVCs, etc.). The results show that for all the investigated datasets, RH is the predominant determining factor (Fig. S3B). At given RH levels, q_w of inorganic species typically varied within 1.5 times. This can be largely explained by the similar q_w – RH curves of $(NH_4)_2SO_4$ and NH_4NO_3 (blue and red lines in Fig. S3B). As inorganic aerosols of urban areas are typically dominated by these two species, variations in other chemical compositions would be of minor influence.

The organics, especially water-soluble organic carbon (WSOC), may also contribute to the L_w. In urban areas, the contribution is usually minor, varying from negligible to ~ 20% [30,39,63]. Only in clean forest areas with very high organic contributions (~67%)[64], the reported contribution could reach ~ 35% [28]. Taking 50% as an upper limit (i.e., L_w could vary by 1.5 times due to the contribution of organics), and combining the typical q_w variations of 1.5 times, the L_w variation due to chemical profile variations is estimated to be 2.25 times. As pK_{AN} is proportional to $\log(L_w{}^2)$, that would correspond to a pK_{AN} variation of ~ 0.7 unit. Again, that is much smaller than that due to meteorological variations (up to ~ 12 units; Fig. 2A).

S2.3 Integrated pK$_{AN}$ uncertainties associated with γ$_{AN}$ and L$_w$

The pK_{AN} is proportional to $\log(L_w{}^2/\gamma^2{}_{AN})$ (Eq. 1b). To quantify the pK_{AN} uncertainties due to γ_{AN} and L_w variations, we introduced the normalized variation term σ_{chem} as:

$$\sigma_{\text{chem}} = (\frac{q_w}{q_w^{\circ}})^2/(\frac{\gamma_{AN}^2}{(\gamma_{AN}^2)^{\circ}})$$

where $q_w°$ is the average reference q_w defined as the average q_w of $(NH_4)_2SO_4$ and NH_4NO_3 at given RH levels (Fig. S3B), and the $(\gamma^2{}_{AN})°$ is the reference $\gamma^2{}_{AN}$ of 0.05, as adopted in Fig. 2 in the main text.

In agreement with the variation patterns of $\gamma^2{}_{AN}$ and $L_w{}^2$ as discussed above (SI text S2.1-S2.2), for all the datasets investigated, the σ_{chem} is within 1 unit. That is, pK_{AN} variations due to chemical profiles through changes in γ_{AN} and L_w are typically within 1 unit. Correspondingly, the pK_{AN} uncertainty is set to be ± 0.5 unit in the following sensitivity tests (SI sect. S2.4).

The σ_{chem} variation range of within 1 unit is far less than pK_{AN} variations due to meteorological variations (up to ~ 12 units; Fig. 2A). Therefore, in discussing the large-scale spatiotemporal variations when the meteorology can change much, pK_{AN} is largely determined by the synoptic conditions. In comparison, when discussing specific conditions with less L_w and temperature fluctuations (especially under lab conditions, etc), changes in $\gamma^2{}_{AN}$ and q_w can be important.

S2.4 Influence of pK$_{AN}$ uncertainties on nitrate control regime classifications

In the A_{ex} - pK_{AN} plots like Figs. 2B-D, the pK_{AN} uncertainties would shift the points along the x axis. Correspondingly, for points close to the regime boundary lines, the regimes may change due to the pK_{AN} uncertainties. Potential regime transitions may occur between $HNO_3{}^T$-Trans regimes, $NH_3{}^T$-Trans regimes, and the Meteo regime and all other regimes. For instance, when pK_{AN} is varied by ± 0.5 unit, the SE-US scenario may convert from the Meteo regime into the

HNO_3^T regime, while regimes for all the other three scenarios (NE-US, YRD and NCP) would be kept the same (Fig. S3D).

We further examined the influence of pK_{AN} uncertainties on nitrate control regime distributions with sensitivity analysis for both the GEOS-Chem and WRF-CMAQ simulations. Fig. S3 E, F show regions that experienced regime changes when pK_{AN} is varied by ± 0.5 unit. Globally, the most susceptible regions are at the boundary of Meteo and other regimes, i.e. regions with higher pK_{AN} levels (Fig. S3E). In comparison, susceptible regions in China belong largely to the HNO_3^T-Trans regimes. These regions are characterized with relatively small A_{ex} levels, and are relatively scattered (Fig. S3F).

S3. Numerical derivation of $f_{p,NO3}$ and the nitrate sensitivities to different chemical species

S3.1 Derivation of multiphase dissociation equilibrium of ammonium nitrate

Establishing the equation sets.

For a given aerosol system, the total concentration for each species and the meteorological conditions are known, while the gas-particle partitioning of the volatile species is unknown. To determine the nitrate gas-particle partitioning, the following constraints must be satisfied:

(1) Acid-dissociation equilibrium and gas-particle equilibrium.

The multiphase buffer theory provides a quantitative explanation of how the acidity, gas-particle partitioning and acid dissociation equilibrium are inter-correlated for volatile acids and bases in a multiphase aerosol system. Applying this theory to the multiphase NH_4^+/NH_3 and HNO_3/NO_3^- systems, respectively, we have:

$$K^{*,ni}_{a,NH3} = \frac{[H^+(aq)][NH_3(g)]}{[NH_4^+(aq)]} = \frac{\gamma_{NH4+}}{\gamma_{H+}} K_{a,NH3} \frac{\rho_w}{H_{NH3}\; R\; T\; L_w} \quad (S1)$$

$$K^{*,ni}_{a,HNO3} = \frac{[H^+(aq)][NO_3^-(aq)]}{[HNO_3(g)]} = K_{a,HNO3} \frac{H_{HNO3}\; R\; T\; L_w}{\gamma_{H+}\gamma_{NO3-}\rho_w} \quad (S2)$$

where $K_{a,i}$ and H_i are the molality-based acid dissociation equilibrium constant and Henry's law constant (mol kg^{-1} atm^{-1}) of species i, respectively (see Table S2). T is temperature in K, R is the gas constant of 0.08205 atm L mol^{-1} K^{-1}, ρ_w is water density being 10^{12} μg m^{-3}, L_w is the aerosol water content in μg m^{-3} air, and γ_i is the activity coefficient for species i .

Dividing Eq. S2 by S1, we can eliminate the influence of aerosol acidity (i.e., $[H^+]$), and directly establish the relationship between HNO_3 and NH_3 gas-particle partitioning as:

$$K_{AN} = \frac{K^{*,ni}_{a,HNO_3}}{K^{*,ni}_{a,NH_3}} = \frac{[NO_3^-(aq)]}{[HNO_3(g)]}\frac{[NH_4^+(aq)]}{[NH_3(g)]} = \frac{K_{a,HNO3}H_{HNO3}H_{NH3}}{K_{a,NH3}}\left(\frac{R\ T\ L_w}{\rho_w \gamma_{AN}}\right)^2 \qquad (S3)$$

where γ_{AN} is the activity coefficient of ammonium nitrate defined as $(\gamma_{NO3-}\gamma_{NH4+})^{0.5}$. This is the Eq. 1b in the main text, and represents the overall relationship of acid dissociation equilibrium and gas-particle partitioning for the HNO_3 and NH_3 systems.

(2) Mass conservation.

The mass conservation of HNO_3 and NH_3 would respectively require that:

$$[NH_4^+(aq)] + [NH_3(g)] = [NH_3]^T = \text{constant} \qquad (S4)$$

$$[NO_3^-(aq)] + [HNO_3(g)] = [HNO_3]^T = \text{constant} \qquad (S5)$$

(3) Liquid phase charge balance.

For typical non-coastal urban ambient conditions and coastal urban areas or periods with significant chloride depletions [65,66] alike, the contribution of Cl^- to total anions is usually negligible (< 10% here; see Fig. S3 for details). Under these conditions, the aqueous-phase charge balance can be expressed as:

$$[NH_4^+(aq)] + [NVCs(aq)] = 2[SO_4^{2-}(aq)] + [NO_3^-(aq)] \qquad (S6)$$

Recall that $[NVCs(aq)] = 2\,[Ca^{2+}(aq)] + 2\,[Mg^{2+}(aq)] + [K^+(aq)] + [Na^+(aq)]$ (Eq. 2c). Here we define the free anions from sulfate available for ammonium as:

$$[SO_4^{2-}{}_f] = 2[SO_4^{2-}(aq)] - [NVCs(aq)] = [NH_4^+(aq)] - [NO_3^-(aq)] = \text{constant} \qquad (S7)$$

Then Eq. S6 can be rewritten as:

$$[NH_4^+(aq)] = [NO_3^-(aq)] + [SO_4^{2-}{}_f] \qquad (S8)$$

Based on the above four equations (Eqs. M3-M5, M8), the four unknowns (i.e., $[NH_3(aq)]$, $[NH_3(g)]$, $[NO_3^-(aq)]$ and $[HNO_3(g)]$) can therefore be solved for a given system.

Normalization

To further simplify the expression, we scale the system by $[HNO_3]^T$, and the corresponding variables would be converted into:

$$f_{p,\,NO3} = [NO_3^-(aq)] / [HNO_3^T] \qquad (S9a)$$

$$R_{A/N} = [NH_3^T] / [HNO_3^T] \qquad (S9b)$$

$$S_f = [SO_4^{2-}{}_f] / [HNO_3^{\ T}] = (2[SO_4^{2-}(aq)] - [NVCs(aq)]) / [HNO_3^T] \qquad (S9c)$$

Based on Eqs. M4, M5 and M8, we have:

$$[NH_4^+(aq)] / [HNO_3{}^T] = ([NO_3^-(aq)] + [SO_4^{2-}{}_f]) / [HNO_3]^T = f_{p,\,NO3} + S_f \qquad (S10a)$$

$$[NH_3\,(g)] / [HNO_3{}^T] = ([NH_3{}^T] - [NH_4^+(aq)]) / [HNO_3]^T = R_{A/N} - S_f - f_{p,\,NO3} \qquad (S10b)$$

$$[HNO_3(g)] / [HNO_3{}^T] = ([HNO_3{}^T] - [NO_3^-(aq)]) / [HNO_3]^T = 1 - f_{p,\,NO3} \qquad (S10c)$$

Substituting the above expressions into Eq. S3, we have:

$$K_{AN} = \frac{[NO_3^-(aq)]}{[HNO_3(g)]} \bullet \frac{[NH_4^+(aq)]}{[NH_3(g)]} = \frac{f_{p,NO3}}{(1 - f_{p,NO3})} \bullet \frac{(f_{p,NO3} + S_f)}{(R_{A/N} - S_f - f_{p,NO3})} \qquad (S11)$$

Which is Eq. 2a in the main text (recall that $A_{ex} = R_{A/N} - S_f$). Eq. S11 can be re-arranged into a quadratic equation on the unknown $f_{p,\,NO3}$, and thus the analytical solution of $f_{p,\,NO3}$ can be solved (SI S3). In addition, the sensitivity of nitrate on different variables can be deduced with this equation (see SI S3 for details).

Constraints and applicability

The basic equation for multiphase dissociation equilibrium (Eq. 2a) can be adopted to predict particulate nitrates only when the following criteria are satisfied. (i) Negligible influence of Cl^-, so that Eq. S6 can be applied. Here the "negligible" is defined as the equivalent charge of total chloride is smaller than 10% that of total anions. (ii) equivalent charge of NVCs is smaller than that of total anions, to ensure the participation of ammonia; (iii) equivalent charge of non-volatile anions (i.e., sulfate) is smaller than that of total cations, to ensure the participation of nitrate. These criteria correspond to Eqs. M12a-c of:

$$[HCl^T] / (2[SO_4^{2-}] + [HNO_3{}^T] + [HCl^T]) < 0.1 \qquad (S12a)$$

$$[NVCs(aq)] < 2[SO_4^{2-}] + [HNO_3{}^T] \qquad (S12b)$$

$$2[SO_4^{2-}] < [NVCs(aq)] + [NH_3{}^T] \qquad (S12c)$$

In the symbol systems scaled by the $[HNO_3{}^T]$, M12b, c corresponds to the constraints that:

$$S_f > -1, \text{ and } R_{A/N} > \max(S_f, 0) \qquad (S13)$$

In addition, as all the species of $[NO_3^-(aq)]$, $[NH_4^+(aq)]$, $[HNO_3(g)]$ and $[NH_3\,(g)]$ should be over 0, which requires that the expressions shown in Eq. S9a and S10a-c are all positive. Those correspond to the $f_{p,\,NO3}$ range constraints of:

$$\max(0, -S_f) < f_{p,\,NO3} < \min(A_{ex}, 1) \qquad (S14)$$

This constraint is applied to identify the proper solution of $f_{p,\,NO3}$ (see SI S3.2).

In addition, to enable the gas-particle equilibrium, we confined the application of this framework to fine-mode particles under RH over 40% only, under which conditions the equilibrium timescales are short [1,67,68].

S3.2 Analytical solution of $f_{p,\,NO3}$

The Eq. 2a in the main text of:

$$K_{\mathrm{AN}}(A_{\mathrm{ex}} - f_{\mathrm{p,NO3}}) = \frac{f_{\mathrm{p,NO3}}}{1 - f_{\mathrm{p,NO3}}}(f_{\mathrm{p,NO3}} + S_f) \quad (1a)$$

can be re-arranged into a quadratic equation on the unknown $f_{p,\,NO3}$, which follows the form of

$$\mathrm{A}\, f_{p,\,NO3}^2 + \mathrm{B}\, f_{p,\,NO3} + \mathrm{C} = 0 \quad (S15)$$

Where

$\mathrm{A} = K_{AN} - 1$

$\mathrm{B} = (K_{AN} - 1)\, S_f - K_{AN}(R_{A/N} + 1)$

$\mathrm{C} = K_{AN} A_{ex}$

Combining the constraints described in Eq. S14, we can derive the corresponding solution of $f_{p,\,NO3}$ as:

$$f_{\mathrm{p,\,NO3}} = \frac{A_{\mathrm{ex}}}{R_{\mathrm{A/N}} + 1}, \quad \text{when A = 0, i.e. when } K_{AN} = 1 \quad (S16a)$$

$$f_{\mathrm{p,\,NO3}} = m - \sqrt{m^2 - n}, \text{ when A > 0, i.e., when } K_{AN} > 1 \quad (S16b)$$

$$f_{\mathrm{p,\,NO3}} = m + \sqrt{m^2 - n}, \text{ when A < 0, i.e., when } 0 < K_{AN} < 1 \quad (S16c)$$

$$\text{where } m = -\frac{B}{2A} = 0.5\left(\frac{K_{AN}\left(R_{A/N} + 1\right)}{K_{AN} - 1} - S_f\right)$$

$$n = \frac{C}{A} = \frac{K_{AN} A_{\mathrm{ex}}}{K_{AN} - 1}$$

S3.3 Nitrate sensitivities to different chemical species

Another representation of multiphase dissociation equilibrium equation

To clearly show the relationship among all species of concern, below we define the following symbols to represent all species of concern as:

$$x = [NO_3^-],\ s = [SO_4^{2-}{}_f] = 2[SO_4^{2-}(aq)] - [NVCs(aq)],\ y = [HNO_3^T],\ z = [NH_3^T] \quad (S17)$$

And based on previous discussions, we can derive that (Method M2):

$$[NH_4^+(aq)] = [NO_3^-(aq)] + [SO_4^{2-}{}_f] = x + s \quad (S18a)$$

$$[NH_3(g)] = [NH_3^T] - [NH_4^+(aq)] = z - s - x \quad (S18b)$$

$[HNO_3(g)] = [HNO_3^T] - [NO_3^-] = y - x$ (S18c)

These variables correlate with the $[HNO_3^T]$-scaled ones by:

$f_{p,NO3} = x/y$, $R_{A/N} = z/y$, and $S_f = s/y$ (S19)

In this symbol system, the multiphase dissociation equilibrium can be represented as:

$$K_{AN} = \frac{[NO_3^-(aq)][NH_4^+(aq)]}{[HNO_3(g)][NH_3(g)]} = \frac{x(x+s)}{(y-x)(z-x-s)} \quad (S20)$$

which is another representation of the multiphase dissociation equilibrium equation.

We see that it's hard to represent $[NO_3^-]$ (i.e., x) as a function of other variables, but easier in the other way. Therefore, instead of deriving the $S(X) = \partial[NO_3^-]/\partial[X]$ directly, we firstly derive its reciprocal $S^{-1}(X) = \partial[X]/\partial[NO_3^-] = \partial[X]/\partial x$. We introduce the symbol Y' to represent the partial derivative of variable Y against x hereinafter, namely

$Y' = \partial Y/\partial[NO_3^-] = \partial Y/\partial x$, for any given variable or function Y (S21)

Combining Eqs. S3 and S7, reciprocals of the nitrate sensitivity to species of concern can be represented as:

$S^{-1}([HNO_3^T]) = \partial[HNO_3^T]/\partial[NO_3^-] = y'$ (S22a)

$S^{-1}([NH_3^T]) = \partial[NH_3^T]/\partial[NO_3^-] = z'$ (S22b)

$S^{-1}([SO_4^{2-}(aq)]) = 0.5\ \partial[SO_{4\ f}^{2-}]/\partial[NO_3^-] = 0.5\ s'$ (S22c)

$S^{-1}([NVCs]) = -\ \partial[SO_{4\ f}^{2-}]/\partial[NO_3^-] = -\ s'$ (S22d)

Which can be derived from Eq. 2a. Below we show the derivation of y' and therefore $S(HNO_3^T)$ in detail, while the other variables can be derived analogously.

Derivation of $S(HNO_3^T)$

Based on Eq. S20, we have:

$$y = x + \frac{x(x+s)}{K_{AN}(z-x-s)} \quad (S23)$$

Taking the derivative, we have:

$$y' = 1 + \frac{1}{K_{AN}}\left(\frac{2x+s}{z-x-s} + \frac{x(x+s)}{(z-x-s)^2}\right) = 1 - \frac{1}{K_{AN}} + \frac{A_y(x)}{K_{AN}} \quad (S24)$$

where $A_y(x) = \frac{z(z-s)}{(z-s-x)^2}$

For $A_y(x)$, we divide both the numerator and the denominator by the $[HNO_3^T]^2$, it can be converted into:

$$A_y(x) = \frac{z(z-s)}{(z-x-s)^2} = \frac{(z/y)(z/y-s/y)}{(z/y-s/y-x/y)^2} = \frac{R_{A/N}(R_{A/N}-S_f)}{(R_{A/N}-S_f-f_{p,NO3})^2} \quad (S25)$$

And therefore we have:

$$S([\mathrm{HNO_3^T}]) = (y')^{-1} = 1 - \frac{1}{K_{\mathrm{AN}}} + \frac{R_{\mathrm{A/N}}(R_{\mathrm{A/N}} - S_{\mathrm{f}})}{K_{\mathrm{AN}}(R_{\mathrm{A/N}} - S_{\mathrm{f}} - f_{\mathrm{p,NO3}})^2} \tag{S26}$$

Which is Eq. 4 in the main text. The $f_{\mathrm{p,\,NO3}}$ under current conditions can be solved by Eq. S16, and therefore the value of $S([\mathrm{HNO_3^T}])$ can be derived.

We further tried to express the $S([\mathrm{HNO_3^T}])$ in the chemical species way, which cannot be adopted to solve the value but can help understand the resistance terms. Taking Eqs. S3-S4 into the expression of $A_y(x)$, and considering that z - s = (z – s - x) + x = $[\mathrm{NH_3(g)}] + [\mathrm{NO_3^-(aq)}]$, we see that:

$$\begin{aligned} y' &= 1 - \frac{1}{K_{\mathrm{AN}}}\left(1 - \frac{z(z-s)}{(z-s-x)^2}\right) = 1 - \frac{1}{K_{\mathrm{AN}}}\left(1 - \frac{[\mathrm{NH_3^T}]([\mathrm{NH_3(g)}]+[\mathrm{NO_3^-(aq)}])}{[\mathrm{NH_3(g)}]^2}\right) \\ &= 1 + \frac{1}{K_{\mathrm{AN}}[\mathrm{NH_3(g)}]}\left([\mathrm{NH_4^+(aq)}] + [\mathrm{NO_3^-(aq)}] + \frac{[\mathrm{NH_4^+(aq)}][\mathrm{NO_3^-(aq)}]}{[\mathrm{NH_3(g)}]}\right) \end{aligned} \tag{S27}$$

Further taking the K_{AN} definition of Eq. 1b in, we have:

$$\begin{aligned} y' &= 1 + \frac{[\mathrm{HNO_3(g)}]}{[\mathrm{NO_3^-(aq)}][\mathrm{NH_4^+(aq)}]} \bullet \left([\mathrm{NH_4^+(aq)}] + [\mathrm{NO_3^-(aq)}] + \frac{[\mathrm{NH_4^+(aq)}][\mathrm{NO_3^-(aq)}]}{[\mathrm{NH_3(g)}]}\right) \\ &= 1 + [\mathrm{HNO_3(g)}]\left(\frac{1}{[\mathrm{NO_3^-(aq)}]} + \frac{1}{[\mathrm{NH_4^+(aq)}]} + \frac{1}{[\mathrm{NH_3(g)}]}\right) \\ &= [\mathrm{HNO_3(g)}]\left(\frac{1}{[\mathrm{HNO_3(g)}]} + \frac{1}{[\mathrm{NO_3^-(aq)}]} + \frac{1}{[\mathrm{NH_4^+(aq)}]} + \frac{1}{[\mathrm{NH_3(g)}]}\right) \end{aligned} \tag{S28}$$

Or equivalently,

$$S([\mathrm{HNO_3^T}]) = (y')^{-1} = ([\mathrm{HNO_3(g)}]\, R_{\mathrm{chem}})^{-1} \tag{S29a}$$

where

$$R_{\mathrm{chem}} = [\mathrm{NH_3(g)}]^{-1} + [\mathrm{HNO_3(g)}]^{-1} + [\mathrm{NH_4^+(aq)}]^{-1} + [\mathrm{NO_3^-(aq)}]^{-1} \tag{S29b}$$

Which is the solution expressed in chemical species term in Eq. 4. Later we'll see how it can facilitate our comparison between $S(\mathrm{HNO_3^T})$ and $S(\mathrm{NH_3^T})$.

Derivation of $S(\mathrm{NH_3^T})$

Similar to the derivation process of $S(\mathrm{HNO_3^T})$, based on Eq. S20, we see that:

$$z = \frac{x(x+s)}{K_{\mathrm{AN}}(y-x)} + x + s \tag{S30}$$

Taking the derivative, we have:

$$z' = 1 - \frac{1}{K_{\mathrm{AN}}} + \frac{A_z(x)}{K_{\mathrm{AN}}} \quad \text{where } A_z(x) = \frac{y(y+s)}{(y-x)^2} = \frac{(1+S_f)}{(1-f_{p,NO3})^2} \tag{S31}$$

Which is Eq. 5 in the main text, and again, the $f_{\mathrm{p,\,NO3}}$ can be solved by Eq. S16.

The expression in chemical species can be derived similarly as:

$$
\begin{aligned}
z' &= 1-\frac{1}{K_{\mathrm{AN}}}(1-A_z(x)) \\
&= 1+\frac{[\mathrm{NH_3(g)}][\mathrm{HNO_3^-(g)}]}{[\mathrm{NO_3^-(aq)}][\mathrm{NH_4^+(aq)}]}\left(1-\frac{[\mathrm{HNO_3^T}]([\mathrm{HNO_3^T}]+[\mathrm{NH_4^+(aq)}]-[\mathrm{NO_3^-(aq)}])}{[\mathrm{HNO_3^-(g)}]^2}\right) \\
&= [\mathrm{NH_3(g)}]\left(\frac{1}{[\mathrm{NH_3(g)}]}+\frac{1}{[\mathrm{NH_4^+(aq)}]}+\frac{1}{[\mathrm{NO_3^-(aq)}]}+\frac{1}{[\mathrm{HNO_3^-(g)}]}\right)
\end{aligned} \quad \text{(S32)}
$$

Or equivalently,

$$S([\mathrm{NH_3^T}]) = (z')^{-1} = ([\mathrm{NH_3(g)}]R_{\mathrm{chem}})^{-1} \quad \text{(S33)}$$

Which is the solution expressed in chemical species term in Eq. 5.

Comparing Eq. S29 with Eq. S33, we can simply see that $S(HNO_3^T)$ is equal to $S(NH_3^T)$ when $[NH_3(g)] = [HNO_3(g)]$. Taking the particle-phase charge balance (Eqs. M4 to M6, Eqs. M10b to M10c) into consideration, this would require that the total cations ($[NVCs(aq)] + [NH_3^T]$) equal that of the total anions (i.e., $2[SO_4^{2-}] + [HNO_3^T]$), or $A_{ex} = 1$, which is Eq. 3 in the main text.

Derivation of $S(SO_4^{2-})$ and S(NVCs)

To derive $S(SO_4^{2-})$ and S(NVCs), we first need to derive s'. Based on Eq. S20, we have:

$$s = -x+\frac{K_{\mathrm{AN}}z(y-x)}{K_{\mathrm{AN}}y+(1-K_{\mathrm{AN}})x} \quad \text{(S34)}$$

Therefore we can derive:

$$s' = -1-\frac{K_{\mathrm{AN}}yz}{(K_{\mathrm{AN}}y+(1-K_{\mathrm{AN}})x)^2} = -1-\frac{K_{\mathrm{AN}}R_{A/N}}{(K_{\mathrm{AN}}+(1-K_{\mathrm{AN}})f_{p,NO3})^2} \quad \text{(S35)}$$

Based on which Eqs. 6 and 7 in the main text can be derived.

Similarly, we can try to show the expression in terms of chemical species as:

$$
\begin{aligned}
s' &= -1-\frac{K_{\mathrm{AN}}[\mathrm{HNO_3^T}][\mathrm{NH_3^T}]}{(K_{\mathrm{AN}}[\mathrm{HNO_3^T}]+(1-K_{\mathrm{AN}})[\mathrm{NO_3^-(aq)}])^2} \\
&= -\frac{[\mathrm{NH_3(g)}][\mathrm{NH_4^+(aq)}]}{[\mathrm{NH_3^T}]}R_{chem}
\end{aligned} \quad \text{(S36)}
$$

Based on which the chemical expression term in Eqs. 6-7 can be derived.

S4. Detailed configuration of model simulations

<u>S4.1 GEOS-Chem simulation</u>

We utilized the 3-D global chemical transport model GEOS-Chem [69] v.14.2.3 (https://zenodo.org/records/10246625; last access: 2025/4/1) for 2019, at a spatial resolution of 2.0° latitude × 2.5° longitude with 47 vertical layers. The meteorology field is from NASA/GMAO's MERRA-2 reanalysis meteorological data product [70]. Here we adopted the dry and wet deposition scheme developed by Wang [71] and Luo [72], respectively. All emissions are managed via HEMCO (Harmonized Emissions Component) 3.0 [73], which integrates and regrids multiple emission datasets. Global anthropogenic emissions (including shipping) of NO_x, SO_2, CO, NH_3, BC, OC and VOCs are obtained from the Community Emissions Data System (CEDS) v2 inventory [74], while aircraft emissions are taken from the AEIC inventory [75]. Global fire emissions are provided by the Global Fire Emissions Database version 4 with small fires (GFEDv4.1s, https://surfdrive.surf.nl/files/index.php/s/5y7TdE6ufwpkAW1; last access: 2025/4/1) [76,77]. Additionally, dust [78], sea salt [79], lighting NO_x [80], soil NO_x [81] and biogenic VOCs (MEGAN v2.1 [82]) are computed online within the HEMCO 3.0 [73].

The annual mean GEOS-Chem $PM_{2.5}$ concentrations are assessed against global $PM_{2.5}$ ground observations. Surface $PM_{2.5}$ monitoring data are gathered from various regions, including Canada (https://data-donnees.az.ec.gc.ca/data/air/monitor/national-air-pollution-surveillance-naps-program/Data-Donnees?lang=en/; last access: 2025/4/1), the United States (https://aqs.epa.gov/aqsweb/airdata/download_files.html#Daily/; last access: 2025/4/1), Europe (https://discomap.eea.europa.eu/map/fme/AirQualityExport.htm/; last access: 2025/4/1), and China (https://www.cnemc.cn/; last access: 2025/4/1). Afterwards, for each monitoring site, the modeled $PM_{2.5}$ concentration of the corresponding grid cell was selected for comparison. The modeled $PM_{2.5}$ exhibits a strong correlation with the observations (correlation coefficient R = 0.85), although it tends to slightly underestimate the measured values, with the normalized mean bias (NMB) being –0.22.

The GEOS-Chem simulated particulate sulfate (SO_4^{2-}), nitrate (NO_3^-) and ammonium (NH_4^+) are evaluated against ground observations from seven monitoring networks, including SPARTAN (Surface Particulate Matter Network, https://www.spartan-network.org/; last access: 2025/4/1), CSN (Chemical Speciation Network, https://www.epa.gov/amtic/chemical-speciation-network-csn; last access: 2025/4/1), CASTNET (Clean Air Status and Trends Network, https://www.epa.gov/castnet; last access: 2025/4/1), IMPROVE (Interagency Monitoring of Protected Visual Environments, https://views.cira.colostate.edu/fed/QueryWizard/Default.aspx; last access: 2025/4/1), EMEP (European Monitoring and Evaluation Programme, https://ebas-data.nilu.no/; last access:

2025/4/1), EEA (European Environment Agency, https://eeadmz1-downloads-webapp.azurewebsites.net/; last access: 2025/4/1) and NACMON (National Aerosol Chemical Composition Monitoring Network of China)[83]. Both the simulations and observations are averaged annually, with approximately 500 valid monitoring stations globally for the year 2019, primarily in the U.S., Europe, and China. The modeled concentrations of these three species correlate relatively well with observations, with *R* ranging from 0.91 to 0.97 and NMB values between –0.24 and 0.05.

S4.2. WRF-CMAQ simulation

The WRF-CMAQ model was adopted to simulate the atmospheric chemistry and transport over China in 2013 and 2019. Detailed model configurations and validations are detailed elsewhere [84,85], and we only briefly describe below. The model was configured with WRFv3.9 (http://www.2.mmm.ucar.edu/wrf/users/wrfv3.9/ ; last access: 2025/4/1) and CMAQv5.2 (https://github.com/USEPA/CMAQ/blob/5.2/CCTM/docs/Release_Notes/; last access: 2025/4/1). The simulation domain contains the Chinese mainland and some parts of South and East Asia with a 36 km horizontal resolution. The vertical resolution has 46 sigma levels from the surface to the tropopause and is collapsed into 28 layers by the Meteorology-Chemistry Interface Processor (MCIP). The NCEP/FNL final reanalysis data (https://rda.ucar.edu/datasets/d083002/; last access: 2025/4/1) are used to provide the meteorological initial and boundary conditions. The CMAQ model was configured with CB05 gas-phase mechanism and AERO6 aerosol module. Six heterogeneous reactions on aerosol surfaces, including the uptake of HO_2, O_3, OH, N_2O_5, NO_3, and HNO_3 were incorporated into the CMAQ model [84]. Time-varying chemical lateral boundary conditions are provided by CAM-Chem (https://www.acom.ucar.edu/cam-chem/cam-chem.shtml , last access: 2025/3/25). We used the Multi-resolution Emission Inventory of China model (MEIC) [86] to provide anthropogenic emissions in China, with the anthropogenic emissions beyond the Chinese mainland derived from the MIX Asian emission inventory of 2010. Biogenic emissions were calculated from the Model of Emissions of Gases and Aerosols from Nature 3.1 (MEGANv3.1) [87] and driven by the inter-annual meteorological inputs from the WRF model. Biomass burning emissions were obtained from the Global Fire Emissions Database (GFED) version 4 [77]. In general, the simulated $PM_{2.5}$ and its compositions show good agreement with the in-situ observations obtained from the China National Environmental Monitoring Center (CNEMC, http://www.cnemc.cn/en/; last access: 2025/4/1), and observation data collected from various sources in our previous study [88]. The simulation performance of all major air

pollutants meets the recommended limits suggested by the United States Environmental Protection Agency (USEPA) [89]. Performance in historical air quality simulation of the WRF-CMAQ modeling system has been fully evaluated in our previous studies [85]. See full evaluation results in our previous studies [84,85].

S5. Efficiency of NOx-control on nitrate

The HNO_3 is largely secondary, produced from the precursor NOx. Therefore, although one needs to control NOx emissions to control HNO_3 concentrations, the decreased HNO_3 may not be proportional to reduced NOx emissions. Correspondingly, the response of nitrate to NOx emission control and its response to HNO_3^T are correlated by:

$$\frac{S(\mathrm{NOx})}{S(\mathrm{HNO_3^T})} = \frac{\partial[\mathrm{NO_3^-}]}{\partial[\mathrm{NOx}]} / \left(\frac{\partial[\mathrm{NO_3^-}]}{\partial[\mathrm{HNO_3^T}]}\right) = \frac{\partial[\mathrm{HNO_3^T}]}{\partial[\mathrm{NOx}]} \tag{S37}$$

The accurate representation of $\partial[HNO_3^T]/\partial[NOx]$ requires the in-depth investigation of reactive nitrogen chemistry in the atmosphere, and is an ongoing hot topic. As a first-order estimation, we adopted the nitrogen oxidation ratio, NOR, to represent this sensitivity. The NOR is defined as:

$$\mathrm{NOR} = \frac{[\mathrm{HNO_3^T}]}{[\mathrm{HNO_3^T}] + [\mathrm{NOx}]} \tag{S38}$$

and is widely adopted to roughly indicate the conversion efficiency from NOx to nitric acid (i.e. $\partial[HNO_3^T]/\partial[NOx]$) [37,54-57,59]. Substituting Eq. S38 into Eq. S37, we have:

$$S(\mathrm{NOx}) = \mathrm{NOR} \cdot S(\mathrm{HNO_3^T}) \tag{S39}$$

Similar to the classification of HNO_3^T versus NH_3^T regimes, we can now classify the NOx versus NH_3^T regimes as: (a) NOx-control regime, if $S(NOx) > 0.1$ and $S(NH_3^T) < 0.1$; (b) NH_3^T -control regime, if $S(NOx) < 0.1$ and $S(NH_3^T) > 0.1$; (c) Transition regime, if both $S(NOx)$ and $S(NH_3^T)$ are over 0.1, and (d) Meteo-regime, if both $S(NOx)$ and $S(NH_3^T)$ are below 0.1.

With this definition, we can acquire the NOx - NH_3^T regime distribution globally and in China (Fig. S7). We found that the NOx-control regime largely overlapped with the HNO_3^T-control regime, especially over the area with typical nitrate pollutions (as indicated in Fig. 4). This suggests the dominant effect of ammonia excess, and the nitrate is still more sensitive to NOx even considering the incomplete atmospheric conversions. Globally, there's some regime transitions, e.g. changing from "transition" to "NH_3-control" over northeastern part of U.S., and part of the eastern Europe-North Asia, and changing from "HNO_3^T" regime to "Meteo" regime in Southeast Asia, northwestern and middle-eastern coast of Africa, etc. (Fig. S7A, B)

In comparison, few areas experience regime changes in China (Fig. S7C, D). Nevertheless, the regime distribution in Fig. 4 and our major conclusions would hold, that NOx control is more effective than NH_3 control in regions with typical nitrate pollutions.

S6. Reconciling the debate on nitrate control regimes

While our study shows that most parts of China lie in the HNO_3^T-control regime, some previous studies conclude that the NH_3 control is more effective to control nitrate over NCP and/or China. On second check, we found that the above discrepancy is mainly due to the difference in the definition of sensitivity, as well as the simulated extent of ammonia excess, and the type of efficiency of concern, as detailed below.

First, while the sensitivity defined in our study is based on the absolute molar concentrations with the unit of μmol m^{-3} ΔNO_3^- / μmol m^{-3} ΔX, some other studies are based on relative percentages, with the unit of (% ΔNO_3^- / % ΔX). For example, Zhai et al.[2] compared the effectiveness of NO_3^- control policy based on the reduced NO_3^- percentages (%) in response to 0% ~ 50% of NH_3^T or HNO_3^T reductions. In another study by Gu et al. [17], the contribution of NH_3 versus NOx to $PM_{2.5}$ is estimated by the "Nr share", which is "determined by modeling with and without Nr emission", while the simulation "without Nr emission" is roughly equivalent of reducing 100% of NH_3^T or HNO_3^T. However, as the molar concentration of NH_3^T is typically far higher than that of HNO_3^T, the comparison based on percentage can be misleading. For instance, based on our GEOS-Chem simulations, the molar concentration of NH_3^T is generally 5-10 times that of HNO_3^T over the ammonium-nitrate dominated regions. Thus, the 1% reduction in NH_3^T is actually 5-10 times of the 1% reduction in HNO_3^T. As shown in Fig. S8, the optimized control policy may differ in terms of different definitions of sensitivities, especially for the "transition" regimes. While HNO_3^T control is more effective in terms of absolute concentrations over YRD region (Fig. S8B), the NH_3^T control is more effective in terms of percentage reductions (Fig. S8E).

Second, the extent of ammonia excess may vary with the time period of concern and the model configurations. For example, instead of the A_{ex} as adopted in this study, some previous studies have adopted another indicator, $R = [NH_3^T]/([HNO_3^T]+2[SO_4^{2-}])$, to indicate the extent of ammonia excess, which is roughly comparable with our A_{ex}. As shown in Fig. S9A, in winter 2013 over the NCP region, the estimated R by Zhai et al.[2] based on GEOS-Chem simulations is generally lower than our results based on WRF-CMAQ simulations, with R being ~1 or lower

during polluted conditions (SNA concentration over ~ 60 μg m-3). As the pK_{AN} is usually low in winter NCP (Fig. 2A), the control regime can be highly sensitive to *R* around *R*~1, with quick transition from NH_3^T-regime to HNO_3^T-regime when *R* change from ~0.9 to ~1.1 (Fig. 2E). In comparison, our WRF-CMAQ results suggested an *R* of over 1 under all conditions, and the corresponding regime is nearly always HNO_3^T-regime. In addition, a clear increasing trend is found from 2013 to 2019, which can be largely explained by the decreased sulfate (Fig. S5). Therefore, by the year of 2019, NCP has already come into the HNO_3^T-regime, and NOx control has become far more effective than NH_3 as compared to conditions in 2013.

Third, while here we focused on the chemical efficiency, some other studies focused on the cost-efficiency of targeted measures. For example, Gu et al. [17] combined the chemical transport model with the GAINS model, and found that the marginal abatement cost of ammonia emission is lower than that of nitrogen oxides emission globally. Similarly, Liu et al[16] emphasized the cost-effectiveness of improving agricultural management. In their study, the corresponding $PM_{2.5}$ reduction is actually estimated under a combined pathway of 50% reduction in total NH_3 emission, together with a 15% reduction in SO_2 and NOx emissions. The combined reductions make it hard to distinguish the dominant factor between NH_3 and NOx. The chemical versus cost efficiencies are correlated by the control cost of species X: while reducing per mole of HNO_3^T may reduce more nitrate, the cost associated with that may be larger than the cost to reduce per mole of NH_3^T. The cost estimation is beyond the scope of this study. Nevertheless, here we provided a baseline for the cost-efficiency studies: if the less sensitive species is to be more cost effective, the ratio of cost must compensate the ratio of chemical sensitivities as outlined in our study.

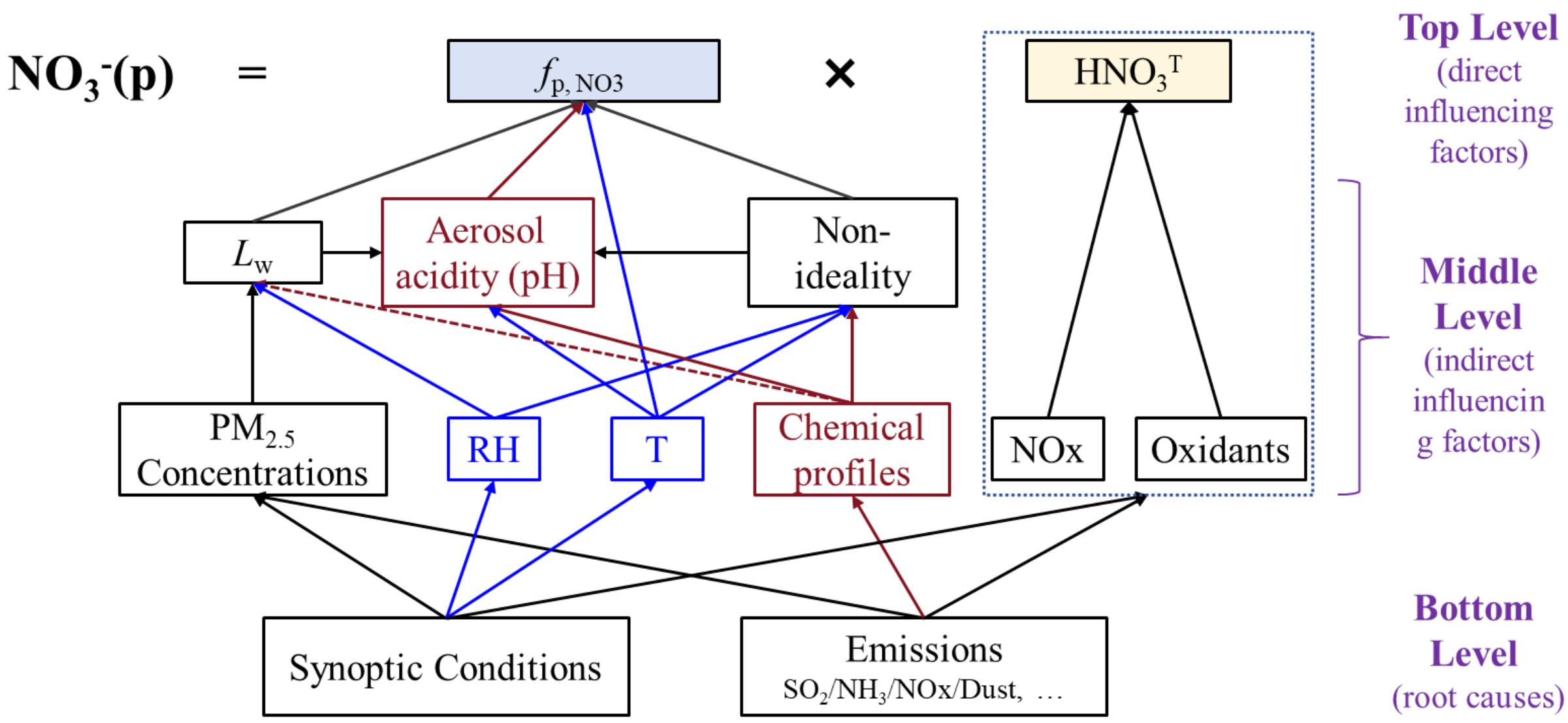

Fig. S1. Interpretative Structural Model (ISM) of the particulate nitrate formation as summarized from previous studies. The ISM approach is widely used to identify and analyze the relationships between factors in complex systems, with the arrows to show the influencing chains. For instance, the $f_{p,\ NO3}$ makes a direct impact on NO_3^- together with HNO_3^T (top-level influencing factors). Among the many influencing factors of $f_{p,\ NO3}$, many studies have suggested that the aerosol pH is a major one, while aerosol pH is further influenced by L_w, temperature, and chemical profiles, etc. (middle-level influencing factors). Fundamentally, variations of these influencing factors are due to variations in synoptic conditions and emissions (bottom level).

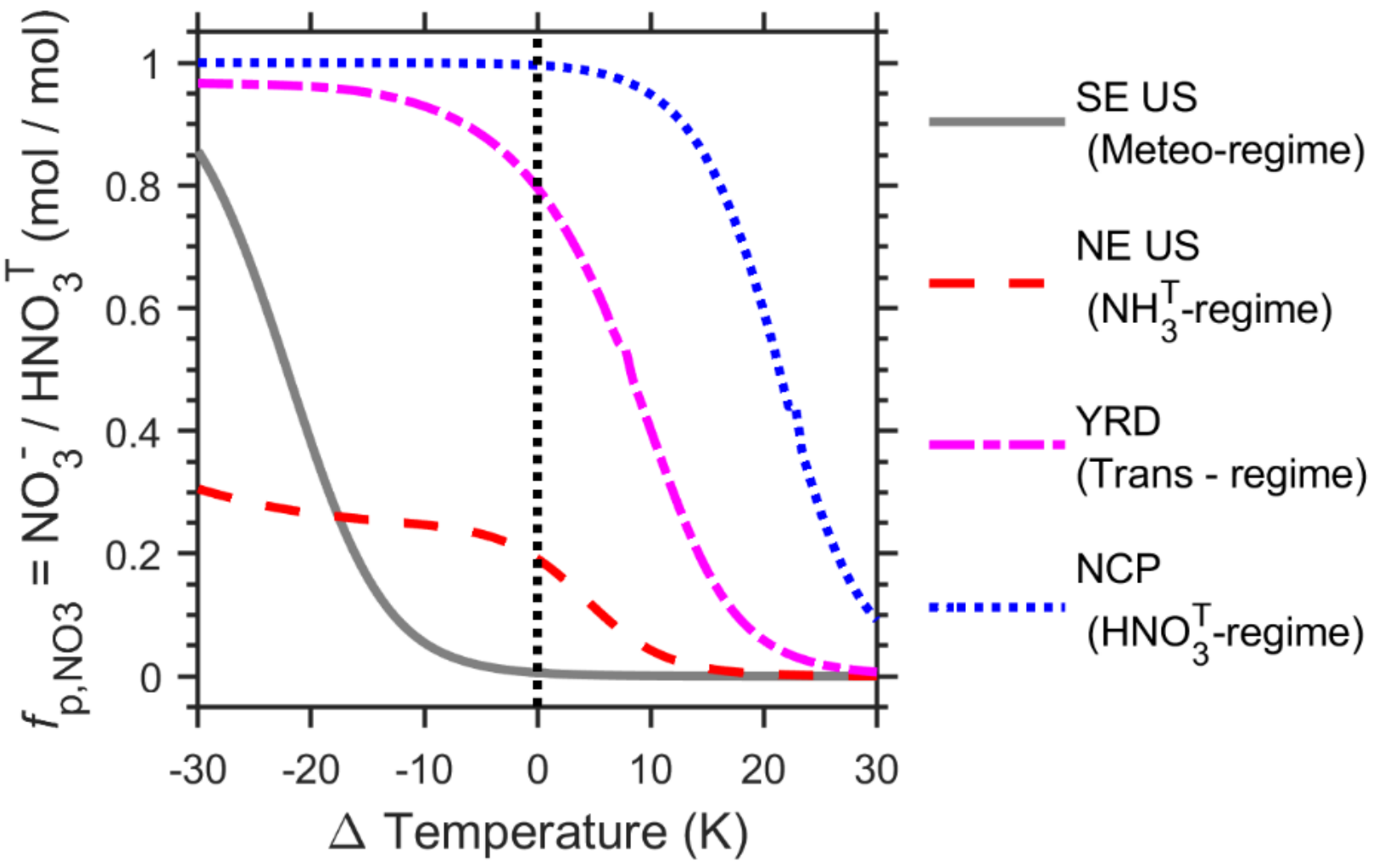

Fig. S2. Influence of temperature on the gas-particle partitioning under different scenarios. The scenarios are the same as shown in Fig. 1 and detailed in Table S1.

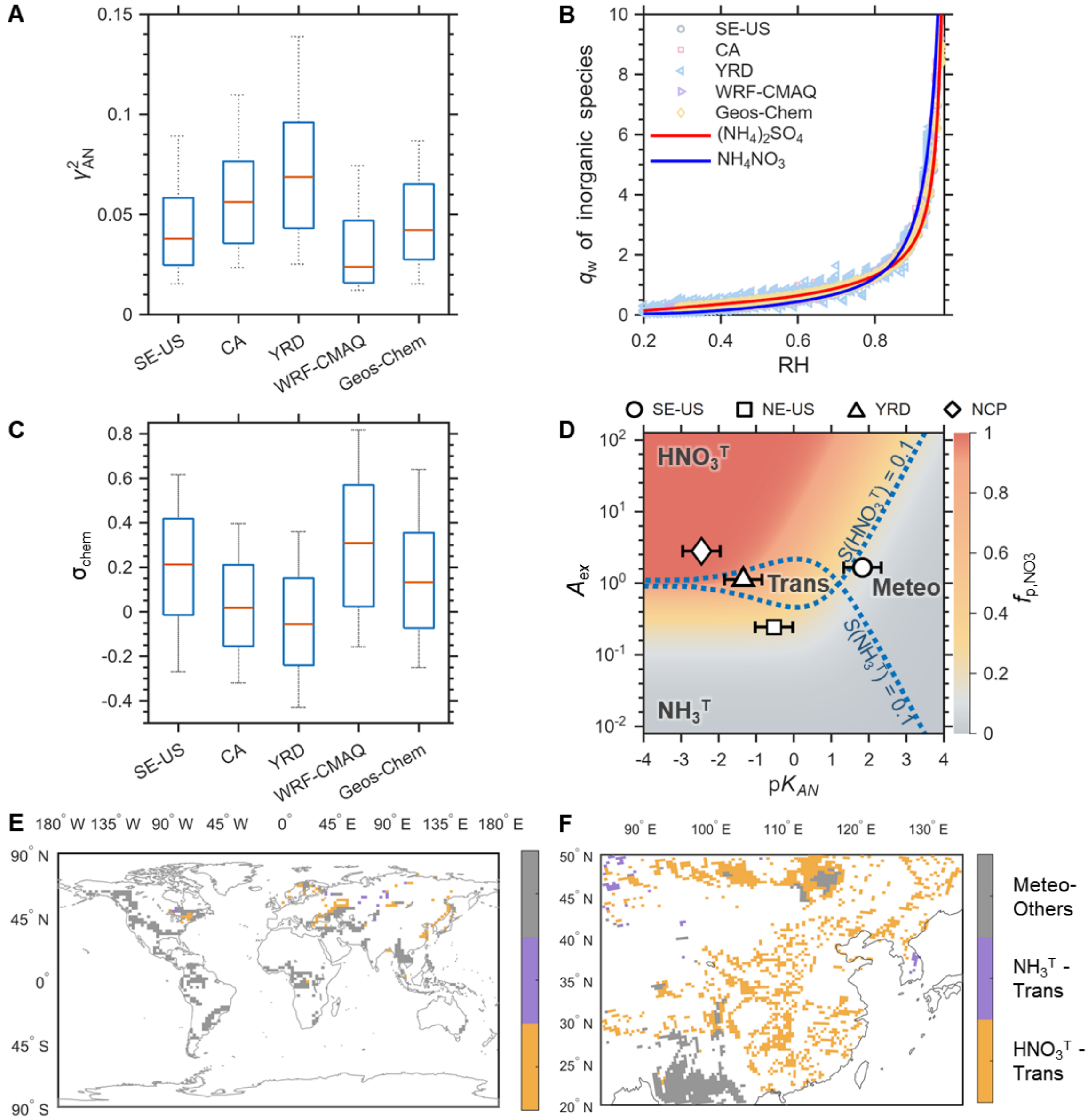

Fig. S3. Estimation of pK_{AN} uncertainties and the corresponding influences on regime classifications. (A) Typical γ^2_{AN} variations ranges. (B) Variations of specific water content q_w with RH for aerosols with different chemical profiles, and for the reference species of pure $(NH_4)_2SO_4$ and NH_4NO_3. (C) Variations of σ_{chem}, which incorporated the influence of both γ^2_{AN} and q_w on pK_{AN}. (D) Same as Fig. 2B, but with the horizontal errorbars of ± 0.5 unit to represent the potential influence of pK_{AN} uncertainty on regime classifications. (E) (F) Distributions of regions with regimes susceptible to a ± 0.5 unit pK_{AN} variations, based on (E) GEOS-Chem simulations and (F) WRF-CMAQ simulations, respectively.

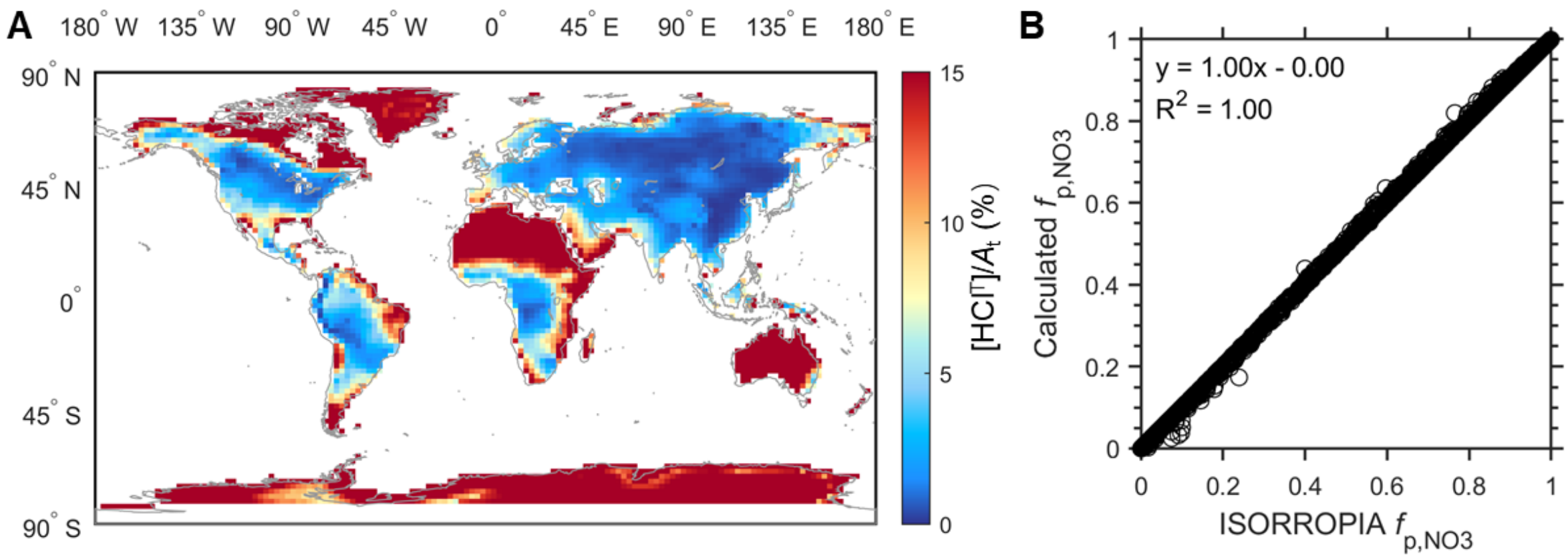

Fig. S4. Influence of total chloride on the applicability of multiphase dissociation equilibrium of ammonium nitrate. (A) Annual average contribution of total chloride to total anions (A_t) in 2019, and (B) comparison of ISORROPIA predicted $f_{p,NO3}$ versus the that calculated from Eq. 1a when [HCl^T]/A_t <10%.

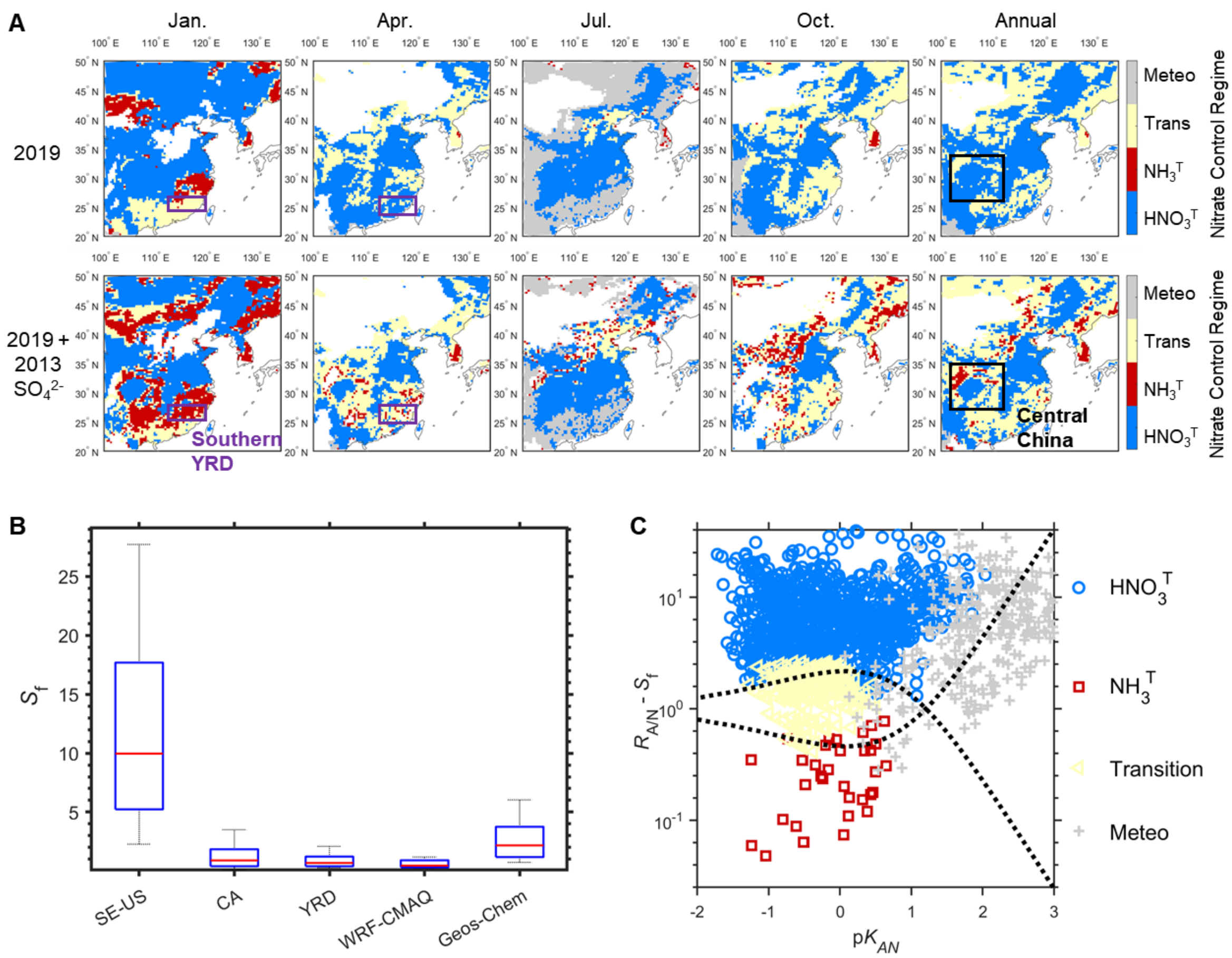

490 **Fig. S5. Influence of sulfate on the nitrate control regime. (A)** Influence through changing the extent of ammonia excess, A_{ex}. The upper panels indicate the nitrate control regimes over eastern and middle part of China in 2019, based on monthly or annual averaged chemical compositions and meteorological conditions. In the lower panels, all the other parameters are kept the same as in the upper panels, while the sulfate is replaced by the corresponding
495 averages in 2013. Reduced sulfate from 2013 to 2019 would elevate A_{ex}, therefore changing the nitrate control regimes**. (B)** Typical variation ranges of S_f based on observation and model simulation dataset. The box and whiskers indicate 10th, 25th, 50th, 75th and 90th percentiles, respectively. The x-axis labels indicate data sources; see SI S2 for details. **(C)** Influence of S_f on the distribution of nitrate control regimes based on the GEOS-Chem simulation results.
500 The dashed lines indicate the regime borders when $S_f = 0$. As the S_f is generally over 0, Meteo-regimes can occur at much lower pK_{AN} levels than the $S_f = 0$ scenario.

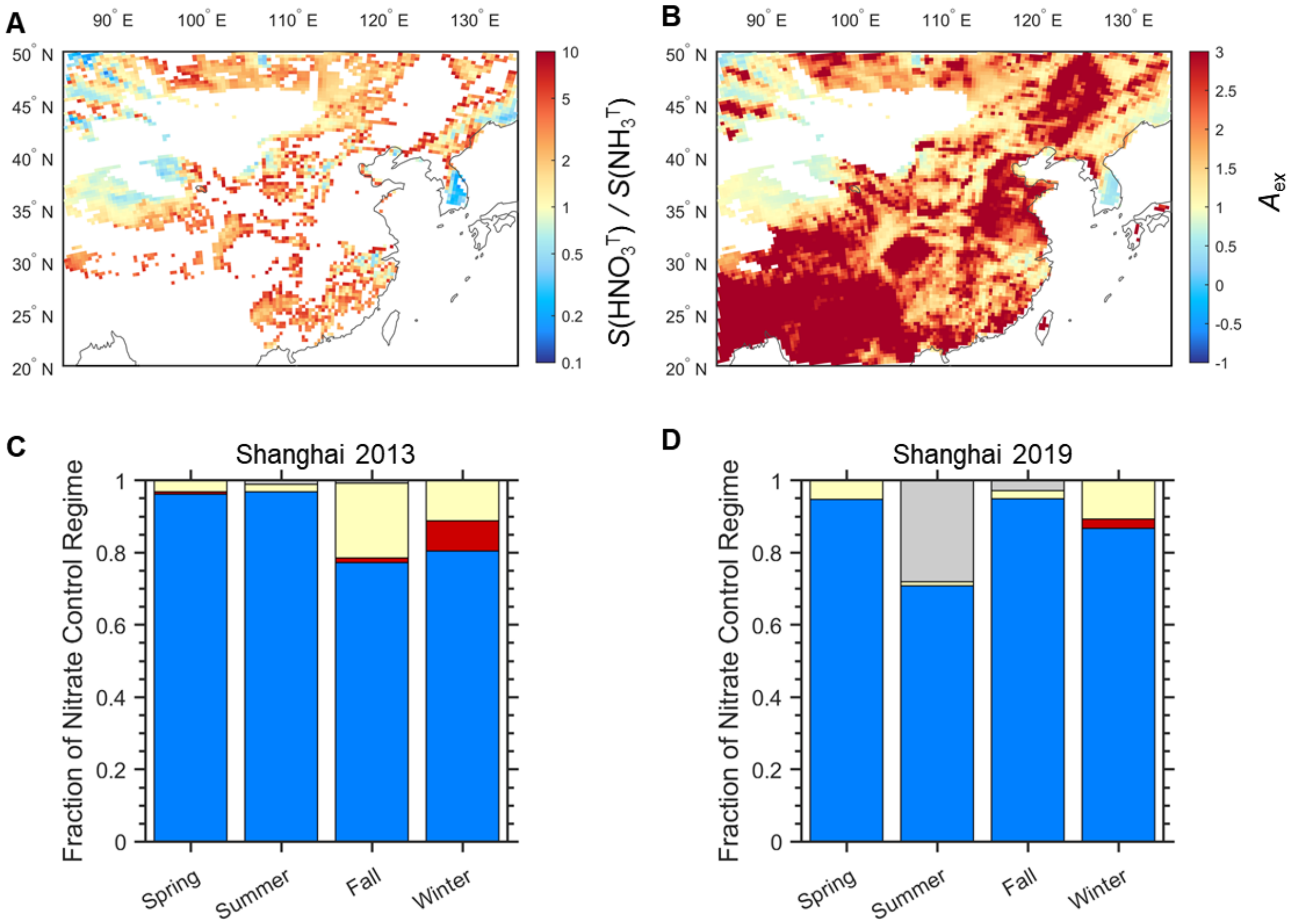

Fig. S6. Dominance of HNO_3^T control regime over China. **(A)** Ratio of nitrate sensitivity to HNO_3^T versus that to NH_3^T in the areas classified as "transition" regime in China, and **(B)** the extent of NH_3 excess in China. **(C-D)** Nitrate-control regime distribution based on Shanghai observations in year 2013 and 2019. The nitrate-control regimes were calculated based on hourly observations, and the percentages indicate the fraction of hours classified as each regime in 2013 and 2019, respectively.

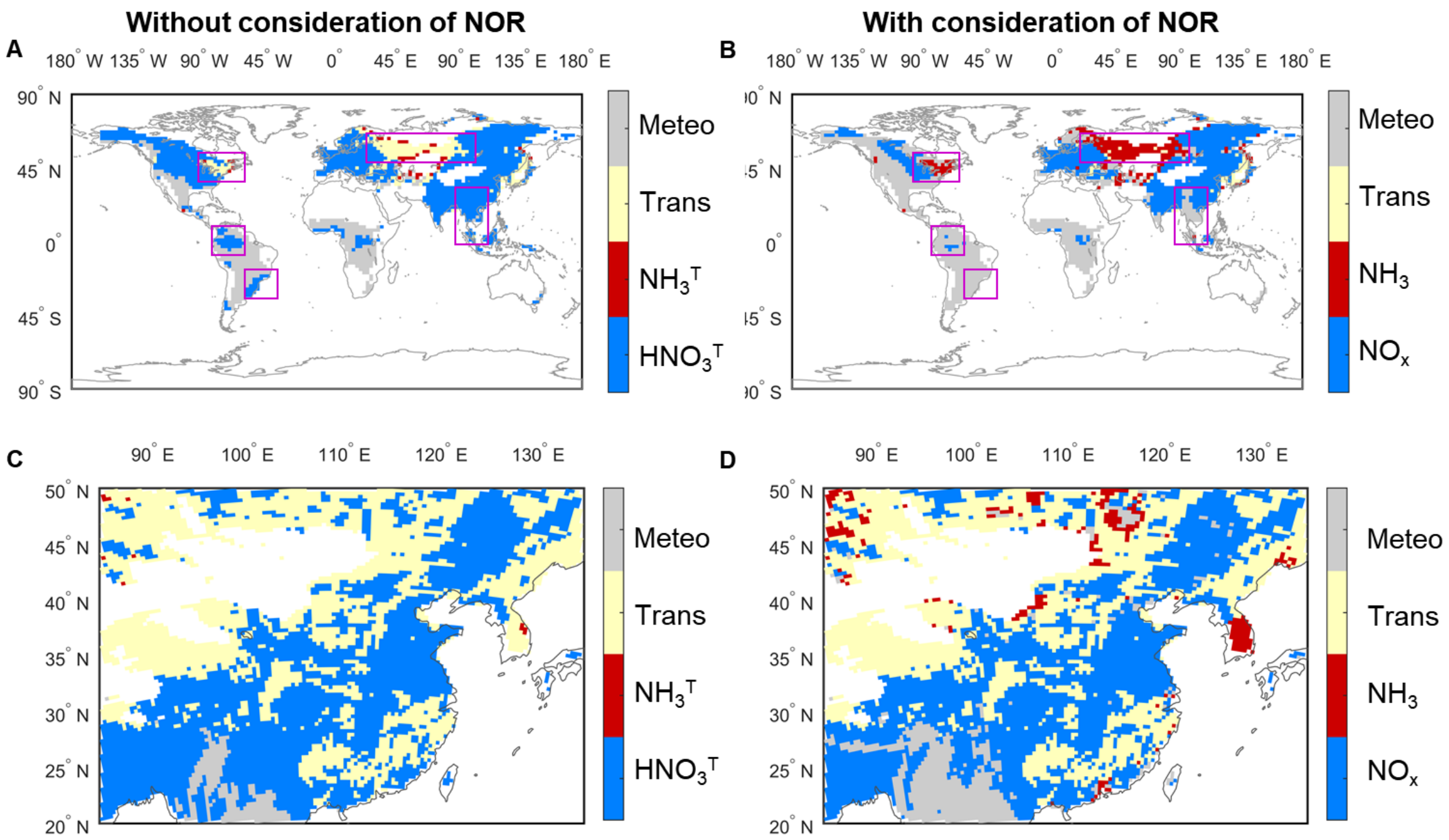

Fig. S7. Comparison of nitrate control regime distributions globally (A, B) and in China (C, D), without (A, C) and with (B, D) consideration of the NOx to HNO_3 conversion ratio. The panels A and C are the same with the Fig. 4 B, D in the main text. The purple squares indicate major areas with regime changes.

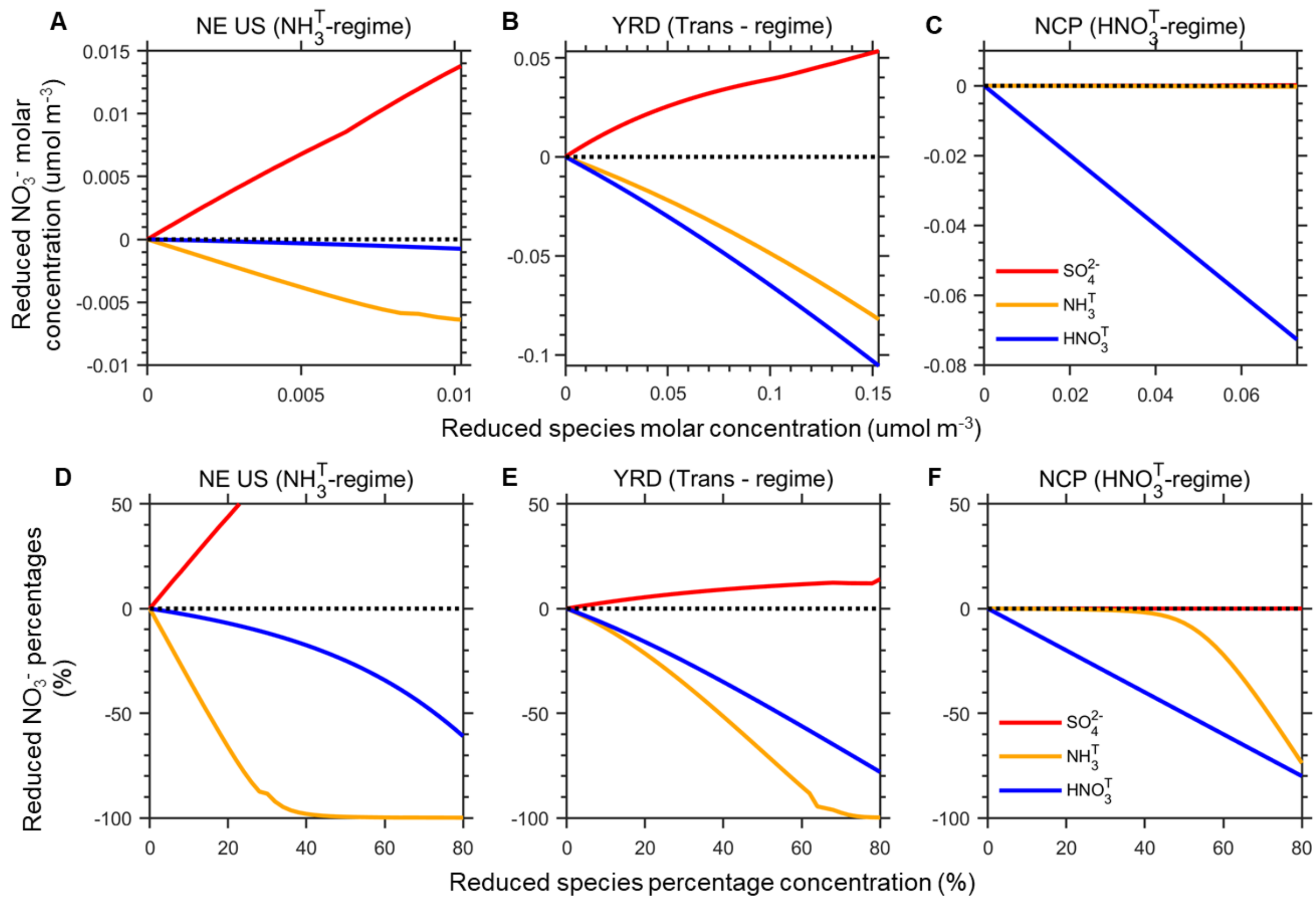

Fig. S8. Influence of sensitivity definition on the control policy selections. Note that for the transition regime YRD, nitrate-control is more effective on the absolute concentration unit (panal B), while ammonia control is more effective judging from the percentage reduction efficiencies (panal D).

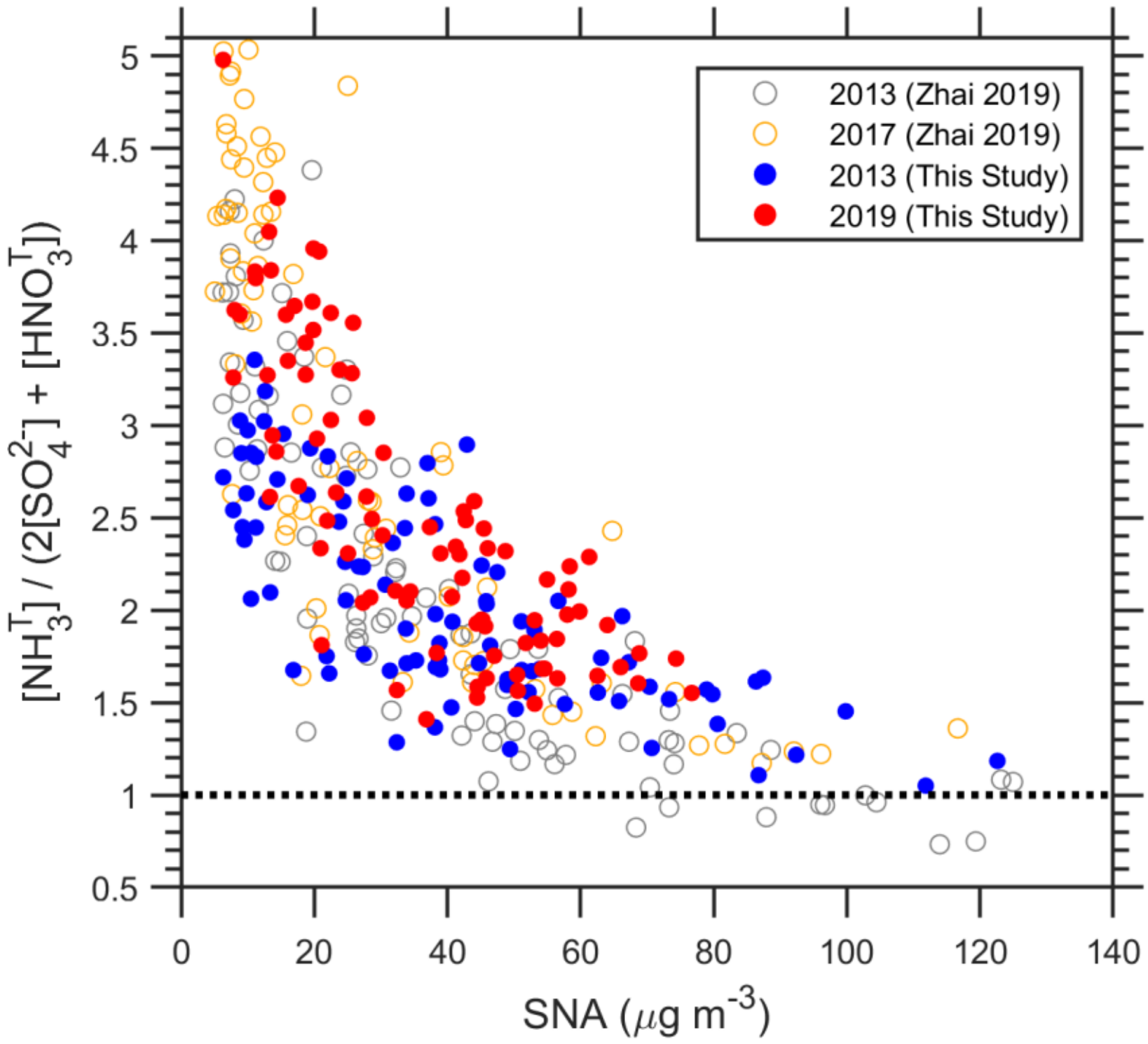

Fig. S9. Comparison of extent of ammonia excess in NCP winter in different simulations. The hollow circles are based on Zhai et al.[2], while the filled circles are based on WRF-CMAQ simulations over the same simulation domain as Zhai et al.[2] in this study.

Table S1. Scenario settings used in manuscript Fig. 1, and supplementary material Fig. S8. All aerosol compositions are in unit of μg m^{-3}.

**Scenario Name**	**Na^+**	**SO_4^{2-}**	**Total NH_3**	**Total HNO_3**	**Total HCl**	**Ca^{2+}**	**K^+**	**Mg^{2+}**	**RH**	***T* (K)**	**Reference**
SE-US[a]	0.03	1.73	0.78	0.45	0.02	0	0	0	0.74	298.15	[28,29]
NE-US[b]	0	1.02	**0.5**	2.21	0	0	0	0	0.58	273.15	[29,30]
YRD[c]	0.01	15.30	11.48	25.87	2.90	1.05	1.43	0.12	0.65	290.71	This study
NCP[d]	0.4	7.3	12.2	14.3	3	0.2	1	0.2	0.52	275.85	[31]

[a] The SE-US scenario represents the average conditions in southeastern U.S. during summer 2013, as detailed in ref. [28,29].

[b] The NE-US scenario represents the average PM_1 compositions and meteorological conditions in northeastern U.S. in winter 2015 based on aircraft measurements, as detailed in ref. [29,30].

[c] The YRD scenario here is based on our measurements, and represents the average conditions during a specific pollution episode from 2013/11/5 to 2013/11/8 in Shanghai.

[d] The NCP scenario here represents the average conditions in Beijing in winter 2017, as detailed in ref. [31].

Table S2. Molality-based equilibrium constants used in calculating the aerosol buffering capacity.

Gas-Aqueous Equilibrium					
Equilibrium	Constant symbol and definition	Constant value (mol kg^{-1} atm^{-1})[a,b]			Ref.
		$K\,(T_0)$	$-\frac{\Delta H_{298}}{RT_0}$	$-\Delta C_{p0}/R$	
NH_3 (g) ⇌ NH_3 (aq)	$H_{NH3} = \frac{\gamma_{NH3(aq)}[NH_3(aq)]}{p_{NH3}}$	57.64	13.79	-5.39	[90]
HNO_3 (g) ⇌ HNO_3 (aq)	$H_{HNO3} = \frac{\gamma_{HNO3(aq)}[HNO_3(aq)]}{p_{HNO3}}$	2.1 (5)	n/a[c]	n/a[c]	[90]
**Aqueous phase Ionization Equilibrium**					
Equilibrium	Constant symbol and definition	Constant value (in mol^2 kg^{-2} for K_w, and in mol kg^{-1} for all others)[a,b]			Ref.
		$K\,(T_0)$	$-\frac{\Delta H_{298}}{RT_0}$	$-\Delta C_{p0}/R$	
H_2O ⇌ H^+ + OH^-	$K_w = \frac{[H^+(aq)][OH^-(aq)]\gamma_{H+(aq)}\gamma_{OH-(aq)}}{a_w}$	1.01 (-14)	-22.52	26.92	[90]
NH_3(aq) + H_2O (aq) ⇌ NH_4^+ + OH^-	$K_{b,NH3} = \frac{[NH_4^+(aq)][OH^-]\gamma_{NH4+(aq)}\gamma_{OH-(aq)}}{[NH_3(aq)]\gamma_{NH3(aq)}a_w}$ c	1.81 (-5)	-1.5	26.92	[90]
HNO_3 ⇌ H^+ + NO_3^-	$K_{a,HNO3} = \frac{[H^+(aq)][NO_3^-(aq)]\gamma_{H+(aq)}\gamma_{NO3-(aq)}}{[HNO_3(aq)]\gamma_{HNO3(aq)}}$	15.4	29.17	16.83	[91]

[a] Read 1.0 (5) as 1.0×10^5.

[b] The equilibrium constant at a specific temperature T is:

$$K(T) = K(T_0)\exp\left[-\frac{\Delta H_{298}}{RT_0}\left(\frac{T_0}{T}-1\right) - \frac{\Delta C_{p0}}{R}\left(1+\ln\left(\frac{T_0}{T}\right)-\frac{T_0}{T}\right)\right]$$

where T_0 = 298.15 K.

[c] The acid dissociation constant of NH_3 applied in manuscript, $K_{a,\,NH3}$, is the ionization equilibrium constant of the conjugate acid of NH_3, and is derived as $K_{a,\,NH3} = K_w/K_{b,\,NH3}$.

Table S3. Information of the long-term observation datasets used in manuscript Fig. 2a, and Figs. S3, S5 and S6.

Scenario Name	Site	Time Span	Reference
SE-US	Centreville, US	2008-2016	[32-34]
CA	Six sites of Canada's National Air Pollution Surveillance (NAPS) Program	2007 - 2016	[35]
India	in South Delhi, India.	2017 - 2018	[36]
NCP	2013 in Beijing,	2013	[37,38]
YRD	Shanghai, China	2013 and 2019	This study; see detailed site and instruments description in [39]

References

1. Seinfeld JH, Pandis SN. *Atmospheric chemistry and physics: from air pollution to climate change*. John Wiley & Sons; 2016.
2. Zhai S, Jacob DJ, Wang X, et al. Control of particulate nitrate air pollution in China. *Nat Geosci* 2021; **14**: 389-395.
3. Gong C, Tian H, Liao H, et al. Global net climate effects of anthropogenic reactive nitrogen. *Nature* 2024; **632**: 557-563.
4. Wang M, Kong W, Marten R, et al. Rapid growth of new atmospheric particles by nitric acid and ammonia condensation. *Nature* 2020; **581**: 184-189.
5. Nenes A, Pandis SN, Kanakidou M, et al. Aerosol acidity and liquid water content regulate the dry deposition of inorganic reactive nitrogen. *Atmos Chem Phys* 2021; **21**: 6023-6033.
6. Xu L, Penner JE. Global simulations of nitrate and ammonium aerosols and their radiative effects. *Atmos Chem Phys* 2012; **12**: 9479-9504.
7. Makkonen R, Romakkaniemi S, Kokkola H, et al. Brightening of the global cloud field by nitric acid and the associated radiative forcing. *Atmospheric Chemistry and Physics* 2012; **12**: 7625-7633.
8. Liu M, Shang F, Lu X, et al. Unexpected response of nitrogen deposition to nitrogen oxide controls and implications for land carbon sink. *Nature Communications* 2022; **13**: 3126.
9. Li HY, Cheng J, Zhang Q, et al. Rapid transition in winter aerosol composition in Beijing from 2014 to 2017: response to clean air actions. *Atmospheric Chemistry and Physics* 2019; **19**: 11485-11499.
10. Wang J, Gao J, Che F, Wang Y, Lin P, Zhang Y. Decade-long trends in chemical component properties of PM2.5 in Beijing, China (2011−2020). *Science of The Total Environment* 2022; **832**: 154664.
11. Qi L, Zheng H, Ding D, Wang S. Responses of sulfate and nitrate to anthropogenic emission changes in eastern China - in perspective of long-term variations. *Science of The Total Environment* 2023; **855**: 158875.
12. Wen L, Chen J, Yang L, et al. Enhanced formation of fine particulate nitrate at a rural site on the North China Plain in summer: The important roles of ammonia and ozone. *Atmospheric Environment* 2015; **101**: 294-302.
13. Wen Z, Xu W, Pan XY, et al. Effects of reactive nitrogen gases on the aerosol formation in Beijing from late autumn to early spring. *Environmental Research Letters* 2021; **16**: 025005.
14. Tian X, Yu H, Wei Y, et al. Gas-particle partitioning process contributes more to nitrate dominated air pollution than oxidation process in northern China. *Aerosol Science and Technology* 2024; **58**: 181-194.
15. Li H, Duan F, Ma Y, et al. Case study of spring haze in Beijing: Characteristics, formation processes, secondary transition, and regional transportation. *Environ Pollut* 2018; **242**: 544-554.
16. Liu M, Huang X, Song Y, et al. Ammonia emission control in China would mitigate haze pollution and nitrogen deposition, but worsen acid rain. *Proceedings of the National Academy of Sciences* 2019; **116**: 7760-7765.
17. Gu B, Zhang L, Van Dingenen R, et al. Abating ammonia is more cost-effective than nitrogen oxides for mitigating PM_{2.5} air pollution. *Science* 2021; **374**: 758-762.

18. Wang Y, Zhang QQ, He K, Zhang Q, Chai L. Sulfate-nitrate-ammonium aerosols over China: response to 2000–2015 emission changes of sulfur dioxide, nitrogen oxides, and ammonia. *Atmos Chem Phys* 2013; **13**: 2635-2652.
19. Xu ZY, Liu MX, Zhang MS, et al. High efficiency of livestock ammonia emission controls in alleviating particulate nitrate during a severe winter haze episode in northern China. *Atmospheric Chemistry and Physics* 2019; **19**: 5605-5613.
20. Guo H, Otjes R, Schlag P, Kiendler-Scharr A, Nenes A, Weber RJ. Effectiveness of ammonia reduction on control of fine particle nitrate. *Atmos Chem Phys* 2018; **18**: 12241-12256.
21. Zang H, Zhao Y, Huo JT, et al. High atmospheric oxidation capacity drives wintertime nitrate pollution in the eastern Yangtze River Delta of China. *Atmospheric Chemistry and Physics* 2022; **22**: 4355-4374.
22. Wei Y, Nenes A, Gao J, et al. Abundant nitrogen oxide and weakly acidic environment synergistically promote daytime particulate nitrate pollution. *Journal of Hazardous Materials* 2023; **456**: 131655.
23. Wen L, Xue L, Dong C, et al. Reduced atmospheric sulfate enhances fine particulate nitrate formation in eastern China. *Science of The Total Environment* 2023; **898**: 165303.
24. Shi X, Nenes A, Xiao Z, et al. High-Resolution Data Sets Unravel the Effects of Sources and Meteorological Conditions on Nitrate and Its Gas-Particle Partitioning. *Environ Sci Technol* 2019; **53**: 3048-3057.
25. Vasilakos P, Russell A, Weber R, Nenes A. Understanding nitrate formation in a world with less sulfate. *Atmos Chem Phys* 2018; **18**: 12765-12775.
26. Duan J, Huang RJ, Li YJ, et al. Summertime and wintertime atmospheric processes of secondary aerosol in Beijing. *Atmospheric Chemistry and Physics* 2020; **20**: 3793-3807.
27. Li Y, Lei L, Sun JX, et al. Significant Reductions in Secondary Aerosols after the Three-Year Action Plan in Beijing Summer. *Environmental Science & Technology* 2023;
28. Guo H, Xu L, Bougiatioti A, et al. Fine-particle water and pH in the southeastern United States. *Atmos Chem Phys* 2015; **15**: 5211.
29. Guo H, Weber RJ, Nenes A. High levels of ammonia do not raise fine particle pH sufficiently to yield nitrogen oxide-dominated sulfate production. *Sci Rep-Uk* 2017; **7**: 12109.
30. Guo H, Sullivan AP, Campuzano-Jost P, et al. Fine particle pH and the partitioning of nitric acid during winter in the northeastern United States. *Journal of Geophysical Research: Atmospheres* 2016; **121**: 10,355-10,376.
31. Ding J, Zhao PS, Su J, Dong Q, Du X, Zhang YF. Aerosol pH and its driving factors in Beijing. *Atmospheric Chemistry and Physics* 2019; **19**: 7939-7954.
32. Edgerton ES, Hartsell BE, Saylor RD, Jansen JJ, Hansen DA, Hidy GM. The Southeastern Aerosol Research and Characterization Study, part 3: Continuous measurements of fine particulate matter mass and composition. *Journal of the Air & Waste Management Association* 2006; **56**: 1325-1341.
33. Weber RJ, Guo H, Russell AG, Nenes A. High aerosol acidity despite declining atmospheric sulfate concentrations over the past 15 years. Letter. *Nature Geosci* 2016; **9**: 282-285.
34. Zheng G, Su H, Cheng Y. Revisiting the Key Driving Processes of the Decadal Trend of Aerosol Acidity in the U.S. *ACS Environmental Au* 2022; **2**: 346-353.
35. Tao Y, Murphy JG. The sensitivity of PM2.5 acidity to meteorological parameters and chemical composition changes: 10-year records from six Canadian monitoring sites. *Atmos Chem Phys* 2019; **19**: 9309-9320.

36. Chen Y, Wang Y, Nenes A, et al. Ammonium chloride associated aerosol liquid water enhances haze in Delhi, India. *Environmental science & technology* 2022; **56**: 7163-7173.
37. Zheng GJ, Duan FK, Su H, et al. Exploring the severe winter haze in Beijing: the impact of synoptic weather, regional transport and heterogeneous reactions. *Atmos Chem Phys* 2015; **15**: 2969-2983.
38. Cheng Y, Zheng G, Wei C, et al. Reactive nitrogen chemistry in aerosol water as a source of sulfate during haze events in China. *Science Advances* 2016; **2**: e1601530.
39. Zhou M, Zheng G, Wang H, et al. Long-term trends and drivers of aerosol pH in eastern China. *Atmos Chem Phys* 2022; **22**: 13833-13844.
40. Fountoukis C, Nenes A. ISORROPIA II: a computationally efficient thermodynamic equilibrium model for K+-Ca2+-Mg2+-NH4+-Na+-SO42--NO3--Cl--H2O aerosols. *Atmos Chem Phys* 2007; **7**: 4639-4659.
41. Nenes A, Pandis SN, Weber RJ, Russell A. Aerosol pH and liquid water content determine when particulate matter is sensitive to ammonia and nitrate availability. *Atmos Chem Phys* 2020; **20**: 3249-3258.
42. Zhao Q, Nenes A, Yu H, et al. Using High-Temporal-Resolution Ambient Data to Investigate Gas-Particle Partitioning of Ammonium over Different Seasons. *Environ Sci Technol* 2020; **54**: 9834-9843.
43. Zheng G, Su H, Wang S, Andreae MO, Pöschl U, Cheng Y. Multiphase buffer theory explains contrasts in atmospheric aerosol acidity. *Science* 2020; **369**: 1374-1377.
44. Karydis VA, Tsimpidi AP, Pozzer A, Lelieveld J. How alkaline compounds control atmospheric aerosol particle acidity. *Atmos Chem Phys* 2021; **21**: 14983-15001.
45. Zheng G, Su H, Wan R, Duan X, Cheng Y. Rising Alkali-to-Acid Ratios in the Atmosphere May Correspond to Increased Aerosol Acidity. *Environmental Science & Technology* 2024; **58**: 16517-16524.
46. Ma N, Zhao WH, Wang WZ, Li XR, Zhou HQ. Large scale of green hydrogen storage: Opportunities and challenges. *Int J Hydrogen Energ* 2024; **50**: 379-396.
47. Zheng G, Su H, Cheng Y. Role of Carbon Dioxide, Ammonia, and Organic Acids in Buffering Atmospheric Acidity: The Distinct Contribution in Clouds and Aerosols. *Environmental Science & Technology* 2023; **57**: 12571-12582.
48. Wang M, Xiao M, Bertozzi B, et al. Synergistic HNO3–H2SO4–NH3 upper tropospheric particle formation. *Nature* 2022; **605**: 483-489.
49. Wang H, Lu K, Tan Z, Chen X, Liu Y, Zhang Y. Formation mechanism and control strategy for particulate nitrate in China. *J Environ Sci (China)* 2023; **123**: 476-486.
50. Xiao HW, Chen TS, Zhang QJ, et al. Changes in the dominant contributions of nitrate formation and sources during haze episodes: Insights from dual isotopic evidence. *Journal of Geophysical Research: Atmospheres* 2025; **130**: e2024JD042175.
51. Zhang Z, Lu B, Liu C, et al. Nitrate pollution deterioration in winter driven by surface ozone increase. *npj Climate and Atmospheric Science* 2024; **7**: 160.
52. Li J, Ho SC, Griffith SM, et al. Concurrent measurements of nitrate at urban and suburban sites identify local nitrate formation as a driver for urban episodic PM2. 5 pollution. *Science of The Total Environment* 2023; **897**: 165351.
53. Chen X, Wang H, Lu K, et al. Field determination of nitrate formation pathway in winter Beijing. *Environmental Science & Technology* 2020; **54**: 9243-9253.
54. Feng X, Chen Y, Chen S, et al. Dominant Contribution of NO3 Radical to NO3– Formation during Heavy Haze Episodes: Insights from High-Time Resolution of Dual Isotopes Δ17O and δ18O. *Environmental Science & Technology* 2023; **57**: 20726-20735.

55. Ma P, Quan J, Dou Y, et al. Regime‐dependence of nocturnal nitrate formation via N2O5 hydrolysis and its implication for mitigating nitrate pollution. *Geophysical Research Letters* 2023; **50**: e2023GL106183.
56. Zhai T, Lu K, Wang H, et al. Elucidate the formation mechanism of particulate nitrate based on direct radical observations in the Yangtze River Delta summer 2019. *Atmospheric Chemistry and Physics* 2023; **23**: 2379-2391.
57. Zhou M, Nie W, Qiao L, et al. Elevated formation of particulate nitrate from N2O5 hydrolysis in the Yangtze River Delta region from 2011 to 2019. *Geophysical Research Letters* 2022; **49**: e2021GL097393.
58. Ren CH, Huang X, Wang ZL, et al. Nonlinear response of nitrate to NO_x reduction in China during the COVID-19 pandemic. *Atmospheric Environment* 2021; **264**: 118715.
59. Zhang Z, Jiang Z, Guan H, Liang Y, Zheng N, Guo W. Isotopic evidence for the high contribution of wintertime photochemistry to particulate nitrate formation in Northern China. *Journal of Geophysical Research: Atmospheres* 2021; **126**: e2021JD035324.
60. Acharja P, Ghude SD, Sinha B, et al. Thermodynamical framework for effective mitigation of high aerosol loading in the Indo-Gangetic Plain during winter. *Sci Rep-Uk* 2023; **13**: 13667.
61. Lawal AS, Guan X, Liu C, et al. Linked Response of Aerosol Acidity and Ammonia to SO2 and NOx Emissions Reductions in the United States. *Environmental Science & Technology* 2018; **52**: 9861-9873.
62. Wan R, Zheng G, Li Y, Duan X, Jiang J, He K. Driving factors for the activity coefficient of atmospheric ammonium nitrate: discrepancies among thermodynamic models and impact on nitrate pollutions. *Atmos Chem Phys* 2026; **26**: 1795-1807.
63. Xu M, Hu B, Zhao SM, Yan GX, Wen TX, Zhao XX. Size-resolved water-soluble organic carbon and its significant contribution to aerosol liquid water. *Science of the Total Environment* 2024; **927**: 172396.
64. Xu L, Guo H, Boyd CM, et al. Effects of anthropogenic emissions on aerosol formation from isoprene and monoterpenes in the southeastern United States. *Proceedings of the National Academy of Sciences* 2015; **112**: 37-42.
65. Braun RA, Dadashazar H, MacDonald AB, et al. Impact of Wildfire Emissions on Chloride and Bromide Depletion in Marine Aerosol Particles. *Environmental Science & Technology* 2017; **51**: 9013-9021.
66. Song AJ, Li K, Yang ZM, Tchinda NT, Du L. Marine Volatile Organic Compounds Promote the Chloride Depletion in Sea Salt Aerosols. *Journal of Geophysical Research-Atmospheres* 2025; **130**: e2025JD043495.
67. Fountoukis C, Nenes A, Sullivan A, et al. Thermodynamic characterization of Mexico City aerosol during MILAGRO 2006. *Atmospheric Chemistry and Physics* 2009; **9**: 2141-2156.
68. Preston TC, Zuend A. Equilibration times in viscous and viscoelastic aerosol particles. *Environ Sci-Atmos* 2022; **2**: 1376-1388.
69. Bey I, Jacob DJ, Yantosca RM, et al. Global modeling of tropospheric chemistry with assimilated meteorology: Model description and evaluation. *Journal of Geophysical Research: Atmospheres* 2001; **106**: 23073-23095.
70. Gelaro R, McCarty W, Suárez MJ, et al. The Modern-Era Retrospective Analysis for Research and Applications, Version 2 (MERRA-2). *Journal of Climate* 2017; **30**: 5419-5454.
71. Wang Y, Jacob DJ, Logan JA. Global simulation of tropospheric O3-NOx-hydrocarbon chemistry: 1. Model formulation. *Journal of Geophysical Research: Atmospheres* 1998; **103**: 10713-10725.

72. Luo G, Yu F, Moch JM. Further improvement of wet process treatments in GEOS-Chem v12.6.0: impact on global distributions of aerosols and aerosol precursors. *Geosci Model Dev* 2020; **13**: 2879-2903.
73. Lin HP, Jacob DJ, Lundgren EW, et al. Harmonized Emissions Component (HEMCO) 3.0 as a versatile emissions component for atmospheric models: application in the GEOS-Chem, NASA GEOS, WRF-GC, CESM2, NOAA GEFS-Aerosol, and NOAA UFS models. *Geoscientific Model Development* 2021; **14**: 5487-5506.
74. McDuffie EE, Smith SJ, O'Rourke P, et al. A global anthropogenic emission inventory of atmospheric pollutants from sector-and fuel-specific sources (1970–2017): an application of the Community Emissions Data System (CEDS). *Earth System Science Data* 2020; **12**: 3413-3442.
75. Simone NW, Stettler MEJ, Barrett SRH. Rapid estimation of global civil aviation emissions with uncertainty quantification. *Transportation Research Part D: Transport and Environment* 2013; **25**: 33-41.
76. Randerson JT, Van Der Werf GR, Giglio L, Collatz GJ, Kasibhatla PS. Global Fire Emissions Database, Version 4.1 (GFEDv4). *ORNL DAAC, Oak Ridge, Tennessee, USA* 2018;
77. van der Werf GR, Randerson JT, Giglio L, et al. Global fire emissions estimates during 1997–2016. *Earth Syst Sci Data* 2017; **9**: 697-720.
78. Fairlie TD, Jacob DJ, Park RJ. The impact of transpacific transport of mineral dust in the United States. *Atmospheric Environment* 2007; **41**: 1251-1266.
79. Jaeglé L, Quinn PK, Bates TS, Alexander B, Lin JT. Global distribution of sea salt aerosols: new constraints from in situ and remote sensing observations. *Atmospheric Chemistry and Physics* 2011; **11**: 3137-3157.
80. Murray LT, Jacob DJ, Logan JA, Hudman RC, Koshak WJ. Optimized regional and interannual variability of lightning in a global chemical transport model constrained by LIS/OTD satellite data. *Journal of Geophysical Research: Atmospheres* 2012; **117**: D20307.
81. Hudman RC, Moore NE, Mebust AK, et al. Steps towards a mechanistic model of global soil nitric oxide emissions: implementation and space based-constraints. *Atmospheric Chemistry and Physics* 2012; **12**: 7779-7795.
82. Guenther AB, Jiang XY, Heald CL, et al. The Model of Emissions of Gases and Aerosols from Nature version 2.1 (MEGAN2.1): an extended and updated framework for modeling biogenic emissions. *Geoscientific Model Development* 2012; **5**: 1471-1492.
83. Dao X, Lin Y-C, Cao F, et al. Introduction to the National Aerosol Chemical Composition Monitoring Network of China: Objectives, Current Status, and Outlook. *B Am Meteorol Soc* 2019; **100**: ES337-ES351.
84. Liu Y, Geng G, Cheng J, et al. Drivers of Increasing Ozone during the Two Phases of Clean Air Actions in China 2013–2020. *Environmental Science & Technology* 2023; **57**: 8954-8964.
85. Geng G, Liu Y, Liu Y, et al. Efficacy of China's clean air actions to tackle PM2.5 pollution between 2013 and 2020. *Nat Geosci* 2024; **17**: 987-994.
86. Li M, Zhang Q, Kurokawa JI, et al. MIX: a mosaic Asian anthropogenic emission inventory under the international collaboration framework of the MICS-Asia and HTAP. *Atmos Chem Phys* 2017; **17**: 935-963.
87. Guenther A, Karl T, Harley P, Wiedinmyer C, Palmer PI, Geron C. Estimates of global terrestrial isoprene emissions using MEGAN (Model of Emissions of Gases and Aerosols from Nature). *Atmos Chem Phys* 2006; **6**: 3181-3210.

88. Liu S, Geng G, Xiao Q, et al. Tracking Daily Concentrations of PM2.5 Chemical Composition in China since 2000. *Environmental Science & Technology* 2022; **56**: 16517-16527.
89. Epa U. Guidance on the use of models and other analyses for demonstrating attainment of air quality goals for ozone, PM2. 5, and regional haze. *US Environmental Protection Agency, Office of Air Quality Planning and Standards* 2007;
90. Kim YP, H. SJ, and Saxena P. Atmospheric Gas-Aerosol Equilibrium I. Thermodynamic Model. *Aerosol Science and Technology* 1993; **19**: 157-181.
91. Haynes WM. *CRC handbook of chemistry and physics*. CRC press; 2014.
